# Living on the edge: Exploring the role of coastal refugia in the Alexander Archipelago of Alaska

**DOI:** 10.1002/ece3.4861

**Published:** 2019-02-01

**Authors:** Yadéeh E. Sawyer, Stephen O. MacDonald, Enrique P. Lessa, Joseph A. Cook

**Affiliations:** ^1^ Department of Biology and Museum of Southwestern Biology University of New Mexico Albuquerque New Mexico; ^2^ Departamento de Ecología y Evolución, Facultad de Ciencias Universidad de la República Montevideo Uruguay

**Keywords:** Coastal Refugia Hypothesis, comparative phylogeography, endemism, islands, small mammals

## Abstract

Although islands are of long‐standing interest to biologists, only a handful of studies have investigated the role of climatic history in shaping evolutionary diversification in high‐latitude archipelagos. In this study of the Alexander Archipelago (AA) of Southeast Alaska, we address the impact of glacial cycles on geographic genetic structure for three mammals co‐distributed along the North Pacific Coast. We examined variation in mitochondrial and nuclear loci for long‐tailed voles (*Microtus longicaudus*), northwestern deermice (*Peromyscus keeni*), and dusky shrews (*Sorex monticola*), and then tested hypotheses derived from Species Distribution Models, reconstructions of paleoshorelines, and island area and isolation. In all three species, we identified paleoendemic clades that likely originated in coastal refugia, a finding consistent with other paleoendemic lineages identified in the region such as ermine. Although there is spatial concordance at the regional level for endemism, finer scale spatial and temporal patterns are less clearly defined. Demographic expansion across the region for these distinctive clades is also evident and highlights the dynamic history of Late Quaternary contraction and expansion that characterizes high‐latitude species.

## 1 INTRODUCTION

Varying isolation, area, and topography make islands of long‐standing interest to studies in evolution, ecology, and conservation biology (Berry, 1986; Fattorini, 2009). Limited connectivity between islands lowers genetic exchange, leading to divergent populations and increased endemism (Adler, 1992; Dobzhansky, 1963; Whittaker, 1998). Because many insular biomes remain understudied, endemism is often poorly documented, yet island biotas likely contribute to global biodiversity more than currently appreciated (Bickford et al., 2007). Tropical oceanic islands have provided key insights into our understanding of diversity, especially in relation to how island area and isolation may shape species richness, community assembly, and patterns of diversification (e.g., Gifford & Larson, 2008; Gillespie, Claridge, & Goodacre, 2008; Hamilton, 1963). But even in tropical island systems, understanding of island composition and genetic relationships is more complex than originally assumed (Filardi & Moyle, 2005). Recently, the various impacts of dynamic geologic events of the Quaternary (2.6 Ma – present), such as changes in sea level, are receiving closer scrutiny in lower latitude archipelagos (e.g., Esselstyn, Timm, & Brown, 2009; Heaney, Walsh, & Peterson, 2005) and along coastal ecosystems (Dolby, Ellingson, Findley, & Jacobs, 2018). Relatively few comparative studies (e.g., Pedreschi, Kelly‐Quinn, Caffrey, O'Grady, & Mariani, 2014; Sota & Nagata, 2008), however, have explicitly investigated the role of climatic history in evolutionary diversification in high‐latitude coastal archipelagos, where dynamic glacial advances potentially restructured entire communities repeatedly.

**Figure 1:**
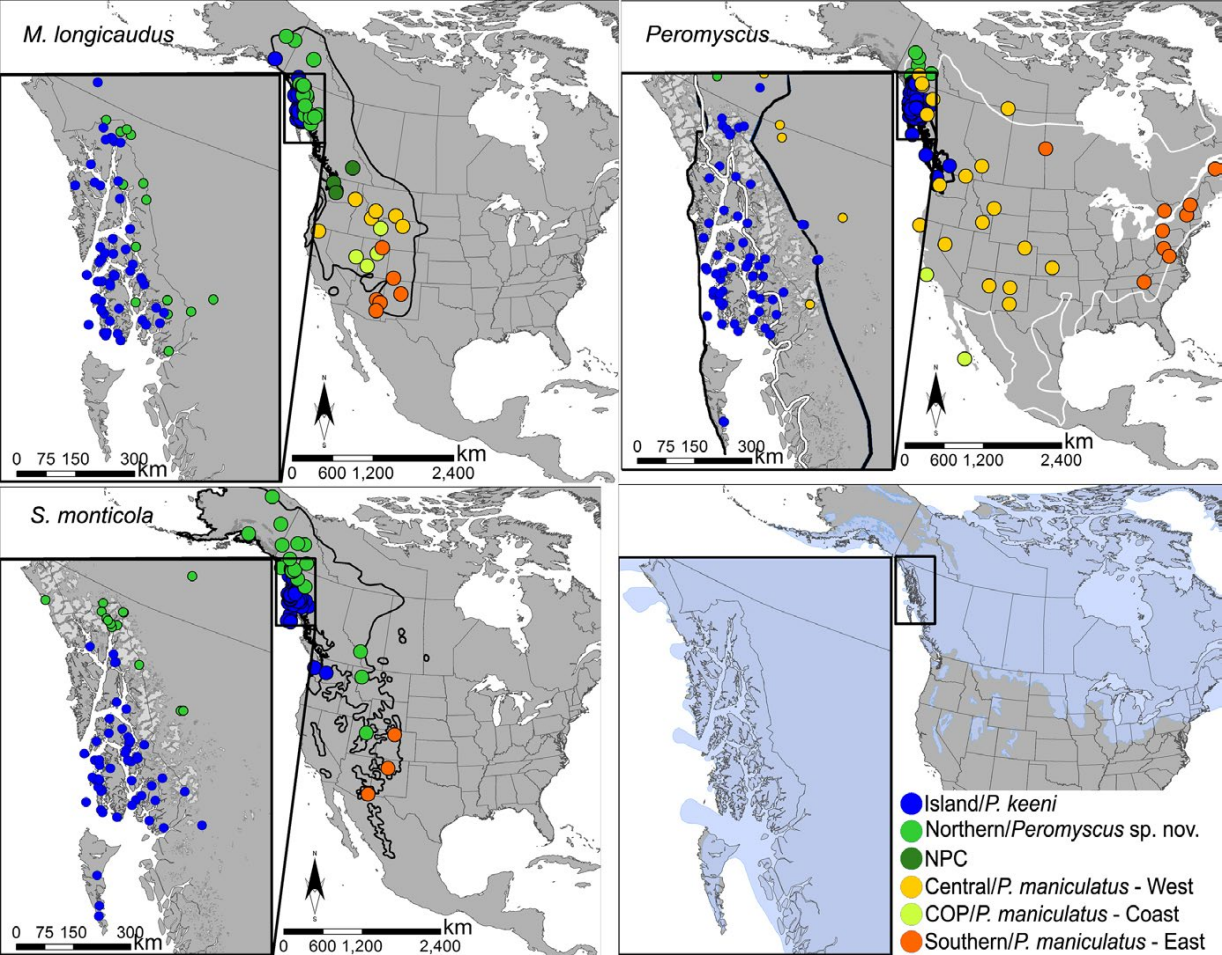
Sampling scheme, range maps, and North American LGM glacial cover. Sampling localities are shown by major cyt*b* lineage. The thick black lines are the current range for each species, with the addition of *P. maniculatus* (white line) on the *Peromyscus* map. The light blue in the bottom right image is LGM glacial ice cover. COP: Colorado Plateau; LGM: Last Glacial Maximum; NPC: North Pacific Coast. Color schemes for species and lineages are held constant across figures

During the Last Glacial Maximum (LGM; between 26.5 and 19 kya), ice covered much of North America (Figure 1; Dyke & Prest, 1987; Mandryk, Josenhans, Fedje, & Mathewes, 2001), restricting species distributions to ice‐free regions in the north (e.g., Beringia), south (continental), or along the coasts (Marr, Allen, & Hebda, 2008). As the Laurentide and Cordilleran ice sheets receded, periglacial populations recolonized previously ice‐covered regions (Eddingsaas, Jacobsen, Lessa, & Cook, 2004; Lessa, Cook, & Patton, 2003) throughout North America. Colonization and extinction dynamics of the land bridge islands along the northwest coast of North America are thought to more closely resemble those of oceanic islands (Conroy, Demboski, & Cook, 1999; Whittaker & Fernández‐Palacios, 2007) because glacial cover from the Cordilleran Ice Sheet hypothetically created a clean slate down to tidewater (Klein, 1965). Areas previously glaciated were presumably colonized from multiple ice‐free (Beringian, southern continental) regions during the late Pleistocene‐early Holocene (14 to 10 kya), with independent recolonization from disparate sources hypothesized to have shaped the contemporary genetic structure of coastal biota. Due to eustatic and isostatic fluxes at the LGM, the Alexander Archipelago (AA) of Alaska and Haida Gwaii of British Columbia experienced sea levels up to 165 m lower (Baichtal, Carlson, & Crockford, 2008; Hetherington et al., 2003; Shugar et al, 2014); however, there remains substantial uncertainty regarding the extent of glaciation in this region (Buma et al., 2013; Carrara, Ager, & Baichtal, 2007; Carrara, Ager, Baichtal, & VanSistine, 2003; Elias, 2013; Fladmark, 1979). The earliest initiation of deglaciation in the region is now estimated at about 17,000 ybp (Lesnek, Briner, Lindqvist, Baichtal, & Heaton, 2018). Although many of the islands were buried under 1,000 m of ice, coastal refugia may have persisted along the exposed western continental shelf (e.g., Baichtal & Carlson, 2010; Fladmark, 1979). This Coastal Refugia Hypothesis remains poorly documented, however, and hinges on whether species persisted and diverged in isolation through the LGM, thereby becoming significant source populations for recolonization of deglaciated island and continental areas in northwestern North America (Byun, Koop, & Reimchen, 1999, 1997; Demboski, Stone, & Cook, 1999).

As glaciers receded and sea levels rose, the islands of the AA became increasingly fragmented, although the sequence of fragmentation is complex due to isostatic rebound (Carrara et al., 2007). Subsequent in situ diversification across the AA hypothetically produced endemic populations due to either long‐term occupation of the region (paleoendemics that originated from coastal refugia) or recent colonization from sources outside the region (neoendemic; Cook, Dawson, & MacDonald, 2006; Cook & MacDonald, 2013). Long‐term field studies (Cook et al., 2017; MacDonald & Cook, 2007) have now produced the density of sampling across the archipelago necessary to begin to explore this complexity for mammals.

The AA is one of the planet's most extensive archipelagos with >1,100 named islands including 7 of the 15 largest United States islands. Together with Haida Gwaii to the south, these archipelagos support a significant portion of the remaining coastal temperate rainforest worldwide (DellaSala et al., 2011; Ecotrust & Conservation International, 1992). Most of the islands within this archipelago are managed by the Tongass National Forest (6.9 million ha; United States Geological Survey, 2010) and have been heavily modified by industrial timber harvests and associated fragmentation (e.g., roads) over the past 60 years (Albert & Schoen, 2013; List, 2000; Schoen & Dovichin, 2007). The rugged and ice‐laden Coast and Wrangell–St. Elias mountain ranges that border the adjacent mainland to the east and north have acted as barriers to dispersal that filtered the movement of species into and out of the region (Cook & MacDonald, 2013).

Previous regional phylogeographic studies identified divergent and endemic populations of various taxa, including a number of vascular and non‐vascular plants (Brodo & Sloan, 2004; Hannon, D'Amore, Witter, & Lamb, 2010; Soltis, Gitzendanner, Strenge, & Soltis, 1997), terrestrial invertebrates (Clarke, Levin, Kavanaugh, & Reimchen, 2001), fishes (Kondzela et al., 1994; O'Reilly, Reimchen, Beech, & Strobeck, 1993; Smith, Nelson, Wood, & Koop, 2001), birds (Barry & Tallmon, 2010; Bull, McCracken, Gaston, Birt, & Friesen, 2010; de Volo, Reynolds, Sonsthagen, Talbot, & Antolin, 2013), and a series of mammals such as northern flying squirrels (*Glaucomys sabrinus*, Bidlack & Cook, 2002), red‐backed voles (genus *Myodes*, Runck, Matocq, & Cook, 2009), red squirrels (*Tamiasciurus hudsonicus*, Hope et al., 2016), ermine (*Mustela erminea*, Dawson, Hope, Talbot, & Cook, 2014; Fleming & Cook, 2002), black bear *(Ursus americanus*, Peacock, Peacock, & Titus, 2007), and mountain goats (*Oreamnos americanus*, Shafer, White, Cote, & Coltman, 2011). To date, few studies in the AA have focused on co‐distributed, multi‐species assemblages.

Small mammals are optimal for exploration of comparative phylogeographic signatures because they are relatively abundant, widespread, and have limited vagility. We examine the role of historical climate variability in structuring contemporary genetic variation of two rodents, *Microtus longicaudus* and *Peromyscus keeni,* and a shrew, *Sorex monticola*; all are widely co‐distributed throughout the AA and adjacent mainland. Previous analyses of mitochondrial DNA variation of a reduced set of localities in the region uncovered significant inter‐population variation in these mammals (Conroy & Cook, 2000; Demboski & Cook, 2001; Lucid & Cook, 2004). Although these species are frequently sympatric, *M. longicaudus* (an herbivore) tends to prefer more open herbaceous habitats, *P. keeni* (an omnivore) typically occurs in forest and scrub habitats, and *S. monticola* (an insectivore) usually occupies forested and non‐forested habitats with dense ground cover (Van Horne, 1981, 1982; Smith & Belk, 1996; Smolen & Keller, 1987; Zheng, Arbogast, & Kenagy, 2003). The dietary isotopic niches of these species largely do not overlap as well (O'Brien, Cook, & Newsome, 2017). If all three species expanded from shared refugia, genetic signatures tracking their expansion histories may be congruent due to the common influence of climatic events (i.e., top‐down environmental control), regardless of specific ecological differences or niche requirements.

**Figure 2:**
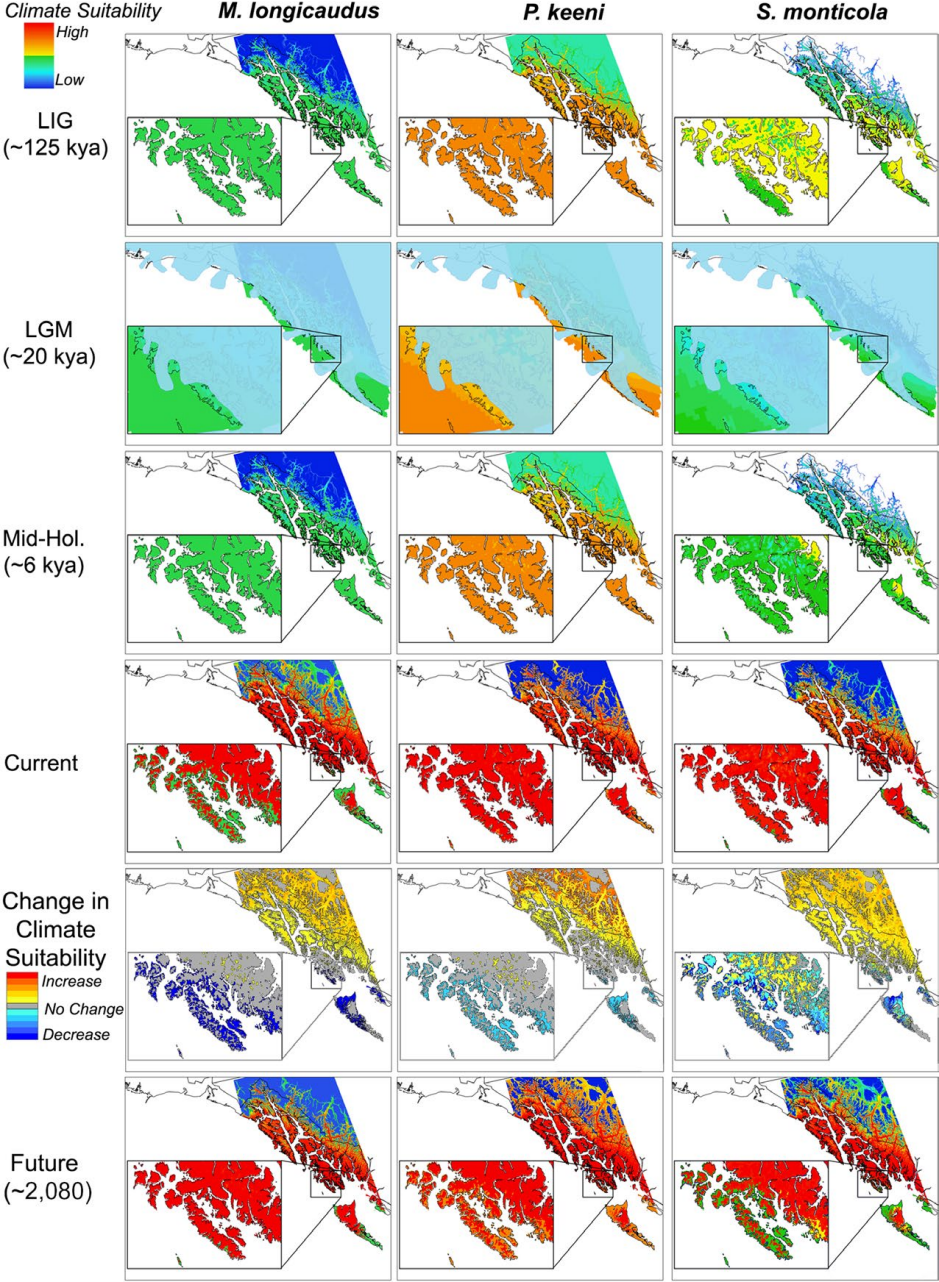
Species distribution models (climate suitability at each time period) for *Microtus longicaudus*, *Peromyscus keeni* and *Sorex monticola* from the Last Inter‐Glacial (LIG), Last Glacial Maximum (LGM; solid blue = glacial ice coverage), Mid‐Holocene (Mid‐Hol.), Current, and Future (approximately the year 2080), including the change in habitat suitability between current and future models

**Figure 3:**
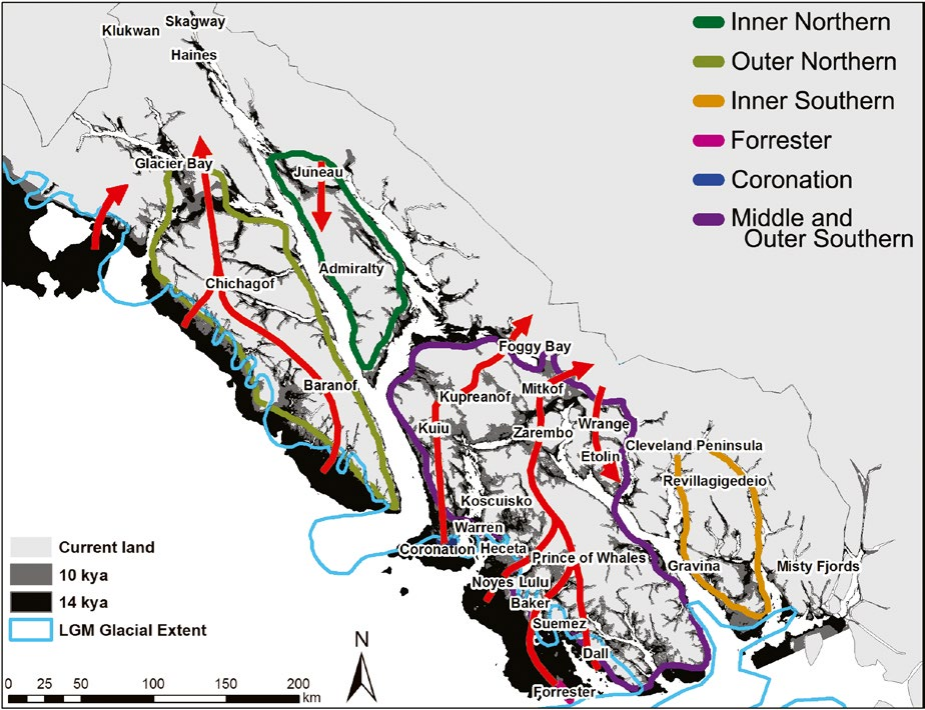
Islands and nearby coastal mainland locations in Alaska, including paleoshorelines (kya = thousands of years ago) and hypothesized island groups (also see Table 2). Red arrows indicate potential colonization across the Alexander Archipelago as a result of change in sea level and glacial cover

In this study, we test three related questions. First, we assess the effects of geological history and climatic conditions on phylogeographic signatures of *M. longicaudus*, *P. keeni*, and *S. monticola*, with a focus on testing the Coastal Refugium Hypothesis (Fladmark, 1979). We use species distribution models (SDMs; Figure 2) and historical bathymetric reconstructions (Figure 3) across species to identify areas of endemism and their spatiotemporal relationship to potential refugia, including coastal refugia.

Next, we test whether phylogeographic signatures are concordant across the three species. An expectation of shared history should result in signatures reflecting similar responses to climatic events and geographic barriers and corridors. Conversely, if taxa have idiosyncratic histories, we expect to observe highly distinctive phylogeographic structure.

Lastly, we integrate the niche models, bathymetry, and demographic analyses to test for concordant signatures of demographic and spatial expansion to assess whether the three species responded similarly to the warming trends of the early Holocene. Signatures of historic expansion may be expected in island populations as a result of post‐glacial colonization following increased island connectivity that occurred when sea levels were lower during glacial periods.

## 2 MATERIALS AND METHODS

### 2.1 Sample collection and sequencing

Specimens (*n* = 137 *M. longicaudus*, 146 *P. keeni*, and 149 *S. monticola*; Supporting Information Appendix S1) were collected through fieldwork between 1991 and 2012 and archived at the University of New Mexico's Museum of Southwestern Biology (MSB) and the University of Alaska Museum of the North. Tissues were also obtained on loan from the University of Washington Burke Museum and Gwaii Haanas National Park Reserve and Haida Heritage Site (13 *P. keeni*, and 3 *S. monticola*). Sampling covered 44 localities across Southeast Alaska and Haida Gwaii. All recognized subspecies (Hall, 1981) found in or near Southeast Alaska for each species were represented. Closely related outgroup species (*n* = 3 *Microtus*, 40 *Peromyscus*, and 9 *Sorex*) were also sequenced. Additionally, we used GenBank sequences representing 41 *M. longicaudus*, and 18 *P. maniculatus* (Supporting Information Appendix S1).

We extracted total genomic DNA to a final concentration of 50 ng/µl using either Omega Bio‐Tek (Norcross, GA) E.Z.N.A. or standard salt extraction (Fleming & Cook, 2002). Polymerase chain reactions (PCR) amplified mitochondrial (mtDNA) cytochrome *b* (cyt *b*) and three nuclear loci per genus, chosen based on variability observed in previous work and preliminary assessment within this study (*Microtus*: Protein C‐est‐2 (ETS2), β‐fibrinogen (FGB), and Recombination Activating Protein 1 (Rag1); *Peromyscus*: β‐fibrinogen (FGB), interphotoreceptor retinoid‐binding protein (IRBP), and zona pellucida 3 (ZP3); *Sorex*: Alcohol Dehydrogenase 2 (ADH2), Apolipoprotein B (ApoB), and β‐fibrinogen (FGB); Supporting Information Appendix S2).

Nuclear heterozygotes were inferred with phase v2.1 (Stephens & Scheet, 2005; Stephens, Smith, & Donnelly, 2001) using five runs with 1,000 iterations (different seeds) and a burn‐in of 1,000. Iterations with the best goodness‐of‐fit were chosen. Posterior probabilities (PP) for nucleotides ≥0.85 were chosen; otherwise ambiguous sites were coded as N. All analyses used phased sequence data. Sequences were edited in Sequencher v4.2 (GeneCodes Corporation), aligned in mega v5.2 (Tamura et al., 2011) using the muscle algorithm and confirmed by eye.

### 2.2 Testing phylogenetic models under the Coastal Refugia Hypothesis

To determine whether climatic conditions in the AA remained within species climatic thresholds, we generated SDMs for each species under current, mid‐Holocene (~6 ka), LGM (~21 kya) (http://pmip2.lsce.ipsl.fr/; Braconnot et al., 2007), last inter‐glacial (LIG; ~120–140 kya), and future conditions (twice the current levels of CO_2_, ~2080, Christensen et al., 2007). Bioclimatic variables were obtained from worldclim (www.worldclim.org, Hijmans, Cameron, Parra, Jones, & Jarvis, 2005) at a resolution of 2.5 arc‐minutes and clipped to incorporate only Southeast Alaska and the surrounding mainland. Test layers were clipped to the extent of sampling, while projection layers were for western North America. enmtools (Warren, Glor, & Turelli, 2008, 2010) was used to determine highly correlated variables (Pearson correlation coefficient ≥0.75), which we then reduced based on those most biologically relevant to small mammals (i.e., temperature related), to avoid over‐parameterized models. Final runs were performed using bioclimatic variables 1 (annual mean temperature), 6 (minimum temperature of coldest month), 7 (temperature annual range), and 11 (mean temperature of coldest quarter), and run using a test percentage of 25%. We obtained species localities from museum databases (e.g., ARCTOS [http://arctos.data-base.uaf.edu] and MaNIS [http://manisnet.org/], Stein & Wieczorek, 2004) in October 2013. Sites with large spatial errors were removed and localities within <12 km of each other were eliminated (Hope et al., 2011) to reduce potential spatial autocorrelation (Reddy & Davalos, 2003), resulting in 127 *M. longicaudus*, 150 *P. keeni*, and 145 *S. monticola* sample localities. SDMs for each species were constructed at each time period using maxent v3.3.3k (Elith et al., 2006; Phillips, Anderson, & Schapire, 2006; Phillips & Dudik, 2008). Final runs were performed using cross‐validation across 10 replicates, with a regularization parameter of 5 (Hope et al., 2011; Warren & Seifert, 2011) and 1,000 iterations. All other values were set as default. Models of LGM were averaged for final results using raster calculator in arcgis 10.1 (ESRI, Redlands, CA, USA). Climate suitability was limited by the low median threshold values over all replicates (Pearson, Raxworthy, Nakamura, & Peterson, 2007). Models were tested for performance using the randomization feature in enmtools.

We estimated potential island refugia, connectivity within and among hypothesized island groups, and potential colonization pathways at different points since the LGM. To re‐create paleoshorelines for three temporal periods: 20 kya (with LGM glacial cover), 14, and 10 kya (Ehlers & Gibbard, 2004), we used information available from Carrara et al., 2003 and Baichtal (pers. com.) in combination with arcgis 10.1 to alter Southeast Alaska to sea levels suggested by estimates of historic sea levels and current bathymetric information (Baichtal & Carlson, 2010; Baichtal pers. Com.). These paleoshoreline reconstructions were then the basis for projected recolonization pathways for these terrestrial mammals.

For this study, AA populations were hypothetically designated as refugial or nonrefugial based on both paleoshoreline reconstructions (Figure 3; Table 1) and climate suitability as determined by the SDMs (Figure 2). Refugia were subaerially exposed, which reflects areas not covered by glacial ice, and not under water (i.e., regions of newly exposed continental shelf). If species persisted in refugia, paleoendemic island populations should show higher divergence than expected for island populations that are the result of recent (Holocene) colonization. Net mtDNA genetic divergences between hypothesized refugial (i.e., persistent) and nonrefugial (i.e., recently colonized) island populations were calculated in mega and standard demographic statistics were calculated for both mtDNA and nuDNA in dnasp for all phased loci to test for varying histories (Table 1).

**Table 1:** Locality information, abbreviations, and island‐specific demographics

Region	Location	Refugia	Hypothesized island group	*M. longicaudus*						
				*n*	*S*	*h*	*Hd*	π	*F*_ST_ (mean)	◊
Alaskan Islands	Admiralty (ADM)*	no	Inner Northern	–	–	–	–	–	–	
	Baker (BKR)*	no	Middle and Outer Southern	–	–	–	–	–	–	
	Baranof (BNF)	no	Outer Northern	–	–	–	–	–	–	
	Chichagof (CGF)*	yes	Outer Northern	5	6	5	1.0000	0.0023	0.2430	
	Coronation (CRN)*	yes	Coronation	4	4	2	0.5000	0.0018	0.3700	◊
	Dall (DAL)*	yes	Middle and Outer Southern	5	9	5	1.0000	0.0033	0.1470	◊
	Etolin (ETN)*	no	Middle and Outer Southern	–	–	–	–	–	–	
	Forrester (FRS)*	yes	Forrester	4	2	2	0.6670	0.0013	0.3960	◊
	Gravina (GRV)*	no	Inner Southern	–	–	–	–	–	–	
	Heceta (HEC)*	no	Middle and Outer Southern	–	–	–	–	–	–	
	Kosciusko (KSC)*	no	Middle and Outer Southern	5	8	4	0.9000	0.0030	0.2080	
	Kuiu (KUI)*	no	Middle and Outer Southern	5	18	5	1.0000	0.0067	0.1600	◊
	Kupreanof (KRF)*	no	Middle and Outer Southern	4	6	3	0.8330	0.0028	0.1950	◊
	Lulu (LUL)*	yes	Middle and Outer Southern	4	0	1	0.0000	0.0000	0.1890	◊
	Mary (MRY)	no	–	–	–	–	–	–	–	
	Mitkof (MIT)*	no	Middle and Outer Southern	–	–	–	–	–	–	
	Noyes (NYS)*	yes	Middle and Outer Southern	3	1	2	0.6670	0.0006	0.1510	◊
	Orr (ORI)	–	–	–	–	–	–	–	–	
	Prince of Wales (POW)*	no	Middle and Outer Southern	6	13	6	1.0000	0.0056	0.1180	◊
	Revillagigedio (REV)*	no	Inner Southern	6	4	4	0.8000	0.0014	0.1730	◊
	San Fernando (SNF)*	no	Middle and Outer Southern	–	–	–	–	–	–	
	Suemez (SMZ)*	yes	Middle and Outer Southern	5	7	4	0.9000	0.0025	0.1360	◊
	Sukkwan (SWN)	–	–	–	–	–	–	–	–	
	Tuxekan (TXN)	no	–	–	–	–	–	–	–	◊
	Warren (WRN)	yes	Middle and Outer Southern	6	1	2	0.3330	0.0003	0.2290	
	Wrangell (WRG)*	no	Middle and Outer Southern	3	3	3	1.0000	0.0018	0.3130	◊
	Zarembo (ZRB)*	no	Middle and Outer Southern	2	4	2	1.0000	0.0035	0.1980	◊
British Columbian Islands	Bischof (BSF)	–	–	–	–	–	–	–	–	–
	Graham (GRM)	–	–	–	–	–	–	–	–	–
	Hotsprings (HTS)	–	–	–	–	–	–	–	–	–
	Moresby (MRB)	–	–	–	–	–	–	–	–	–
	Ramsay (RMS)	–	–	–	–	–	–	–	–	–
	Vancouver (VCR)	–	–	–	–	–	–	–	–	–
Mainland	British Columbia (BC)	no	–	–	–	–	–	–	–	
	Mainland Southeast Alaska (MLSE)	no	–	5	11	5	1.0000	0.0051	0.0735	
	Northern mainalnd Southeast (NMLSE)	no	–	7	14	7	1.0000	0.0053	0.0351	
	Interior Alaska, Yukon and White Pass (North)	no	–	3	9	3	1.0000	0.0058	0.0320	
	Cleveland Peninsula (CP/MLCP)*	–	Inner Southern	–	–	–	–	–	–	
	Foggy Bay (FB/MLFB)*	–	Middle and Outer Southern	–	–	–	–	–	–	
	Glacier Bay (GB/MLGB)*	–	Outer Northern	–	–	–	–	–	–	◊
	Haines (HNS/MLHNS)*	–	–	–	–	–	–	–	–	◊
	Interior Alaska (IAK/MLS)	–	–	–	–	–	–	–	–	
	Juneau (JNO/MLJNO)*	–	Inner Northern	–	–	–	–	–	–	◊
	Klukwan (KLU/MLKLU)	–	–	–	–	–	–	–	–	
	Misty Fjords (MFD/MLMFD)*	–	Inner Southern	–	–	–	–	–	–	◊
	Southeast Central (SEC AK/MLSEC)*	–	–	–	–	–	–	–	–	
	Southeast North (SEN AK/MLSEN)	–	–	–	–	–	–	–	–	
	Southeast South (SES AK/MLSES)	–	–	–	–	–	–	–	–	
	Skagway (SKW/MLSKW)	–	–	–	–	–	–	–	–	
	Taiya River (TYR/MLTYR)	–	–	–	–	–	–	–	–	
	White Pass (WP/MLWP)	–	–	–	–	–	–	–	–	
	British Columbia ‐ Central (BCC)*	–	–	–	–	–	–	–	–	
	British Columbia ‐ North (BCN)	–	–	–	–	–	–	–	–	
	British Columbia ‐ South (BCS)	–	–	–	–	–	–	–	–	
	Washington (WA)	–	–	–	–	–	–	–	–	
	Yukon Territory ‐ Central (YTC)	–	–	–	–	–	–	–	–	
	Yukon Territory ‐ South (YTS)*	–	–	–	–	–	–	–	–	◊
**Region**	**Location**	**Refugia**	**Hypothesized island group**	***P. keeni***						
				***n***	***S***	***h***	***Hd***	**π**	***F*_ST_ (mean)**	◊
Alaskan Islands	Admiralty (ADM)*	no	Inner Northern	4	8	3	0.8330	0.0035	0.1200	
	Baker (BKR)*	no	Middle and Outer Southern	3	5	3	1.0000	0.0029	0.1810	
	Baranof (BNF)	no	Outer Northern	5	4	3	0.7000	0.0014	0.1140	◊
	Chichagof (CGF)*	yes	Outer Northern	4	9	4	1.0000	0.0046	0.1330	◊
	Coronation (CRN)*	yes	Coronation	3	8	3	1.0000	0.0047	0.1760	◊
	Dall (DAL)*	yes	Middle and Outer Southern	6	11	5	0.9330	0.0041	0.1540	◊
	Etolin (ETN)*	no	Middle and Outer Southern	5	4	3	0.7000	0.0017	0.1260	
	Forrester (FRS)*	yes	Forrester	5	3	4	0.9000	0.0011	0.3580	◊
	Gravina (GRV)*	no	Inner Southern	5	2	3	0.7000	0.0007	0.2490	◊
	Heceta (HEC)*	no	Middle and Outer Southern	5	3	3	0.7000	0.0011	0.3040	◊
	Kosciusko (KSC)*	no	Middle and Outer Southern	3	4	2	0.6670	0.0023	0.2990	◊
	Kuiu (KUI)*	no	Middle and Outer Southern	3	6	3	1.0000	0.0035	0.1390	◊
	Kupreanof (KRF)*	no	Middle and Outer Southern	3	3	3	1.0000	0.0017	0.1010	
	Lulu (LUL)*	yes	Middle and Outer Southern	5	3	3	0.7000	0.0012	0.3050	◊
	Mary (MRY)	no	–	2	0	4	1.0000	0.0000	0.4170	◊
	Mitkof (MIT)*	no	Middle and Outer Southern	4	12	4	1.0000	0.0054	0.1000	
	Noyes (NYS)*	yes	Middle and Outer Southern	5	12	5	1.0000	0.0051	0.1650	◊
	Orr (ORI)	–	–	–	–	–	–	–	–	
	Prince of Wales (POW)*	no	Middle and Outer Southern	6	19	6	1.0000	0.0070	0.0692	◊
	Revillagigedio (REV)*	no	Inner Southern	5	20	4	0.9000	0.0076	0.0935	◊
	San Fernando (SNF)*	no	Middle and Outer Southern	4	12	3	0.8330	0.0053	0.2470	◊
	Suemez (SMZ)*	no	Middle and Outer Southern	3	9	3	1.0000	0.0053	0.3510	
	Sukkwan (SWN)	no	–	–	–	–	–	–	–	
	Tuxekan (TXN)	no	–	–	–	–	–	–	–	
	Warren (WRN)	no	Middle and Outer Southern	3	0	1	0.0000	0.0000	0.2620	◊
	Wrangell (WRG)*	no	Middle and Outer Southern	3	6	3	1.0000	0.0035	0.1330	
	Zarembo (ZRB)*	no	Middle and Outer Southern	4	19	4	1.0000	0.0089	0.1310	◊
British Columbian Islands	Bischof (BSF)	no	–	–	–	–	–	–	–	–
	Graham (GRM)	no	–	–	–	–	–	–	–	–
	Hotsprings (HTS)	no	–	–	–	–	–	–	–	◊
	Moresby (MRB)	no	–	–	–	–	–	–	–	–
	Ramsay (RMS)	no	–	–	–	–	–	–	–	◊
	Vancouver (VCR)	no	–	–	–	–	–	–	–	–
Mainland	British Columbia (BC)	no	–	6	20	6	1.0000	0.0066	0.0652	◊
	Mainland Southeast Alaska (MLSE)	no	–	20	25	14	0.9370	0.0044	0.0362	–
	Northern mainalnd Southeast (NMLSE)	no	–	18	24	11	0.8820	0.0052	0.0505	–
	Interior Alaska, Yukon and White Pass (North)	no	–	2	2	2	1.0000	0.0018	0.0390	–
	Cleveland Peninsula (CP/MLCP)*	no	Inner Southern	–	–	–	–	–	–	–
	Foggy Bay (FB/MLFB)*	no	Middle and Outer Southern	–	–	–	–	–	–	–
	Glacier Bay (GB/MLGB)*	no	Outer Northern	–	–	–	–	–	–	–
	Haines (HNS/MLHNS)*	no	–	–	–	–	–	–	–	◊
	Interior Alaska (IAK/MLS)	no	–	–	–	–	–	–	–	–
	Juneau (JNO/MLJNO)*	no	Inner Northern	–	–	–	–	–	–	–
	Klukwan (KLU/MLKLU)	no	–	–	–	–	–	–	–	◊
	Misty Fjords (MFD/MLMFD)*	no	Inner Southern	–	–	–	–	–	–	◊
	Southeast Central (SEC AK/MLSEC)*	no	–	–	–	–	–	–	–	–
	Southeast North (SEN AK/MLSEN)	no	–	–	–	–	–	–	–	–
	Southeast South (SES AK/MLSES)	no	–	–	–	–	–	–	–	–
	Skagway (SKW/MLSKW)	no	–	–	–	–	–	–	–	–
	Taiya River (TYR/MLTYR)	no	–	–	–	–	–	–	–	–
	White Pass (WP/MLWP)	no	–	–	–	–	–	–	–	–
	British Columbia ‐ Central (BCC)*	no	–	–	–	–	–	–	–	–
	British Columbia ‐ North (BCN)	no	–	–	–	–	–	–	–	–
	British Columbia ‐ South (BCS)	no	–	–	–	–	–	–	–	–
	Washington (WA)	no	–	–	–	–	–	–	–	–
	Yukon Territory ‐ Central (YTC)	no	–	–	–	–	–	–	–	◊
	Yukon Territory ‐ South (YTS)*	no	–	–	–	–	–	–	–	◊
**Region**	**Location**	**Refugia**	**Hypothesized island group**	***S. monticola***						
				***n***	***S***	***h***	***Hd***	**π**	***F*_ST_ (mean)**	**◊**
Alaskan Islands	Admiralty (ADM)*	no	Inner Northern	4	3	4	1.0000	0.0013	0.1200	
	Baker (BKR)*	no	Middle and Outer Southern	2	0	1	0.0000	0.0000	0.5470	
	Baranof (BNF)	no	Outer Northern	–	–	–	–	–	–	
	Chichagof (CGF)*	yes	Outer Northern	–	–	–	–	–	–	
	Coronation (CRN)*	yes	Coronation	2	6	2	1.0000	0.0055	0.3450	◊
	Dall (DAL)*	yes	Middle and Outer Southern	5	2	2	0.4000	0.0007	0.1680	
	Etolin (ETN)*	no	Middle and Outer Southern	2	3	2	1.0000	0.0027	0.0273	◊
	Forrester (FRS)*	yes	Forrester	5	3	3	0.7000	0.0013	0.3260	◊
	Gravina (GRV)*	no	Inner Southern	5	5	4	0.9000	0.0019	0.1110	
	Heceta (HEC)*	no	Middle and Outer Southern	4	4	3	0.8330	0.0018	0.3600	◊
	Kosciusko (KSC)*	no	Middle and Outer Southern	3	3	3	1.0000	0.0018	0.1670	
	Kuiu (KUI)*	no	Middle and Outer Southern	3	4	3	1.0000	0.0023	0.0450	
	Kupreanof (KRF)*	no	Middle and Outer Southern	5	3	2	0.4000	0.0011	0.0982	
	Lulu (LUL)*	yes	Middle and Outer Southern	3	4	3	1.0000	0.0023	0.5150	
	Mary (MRY)	no	–	–	–	–	–	–	–	
	Mitkof (MIT)*	no	Middle and Outer Southern	4	1	2	0.6670	0.0006	0.0134	
	Noyes (NYS)*	yes	Middle and Outer Southern	5	8	5	1.0000	0.0028	0.3370	◊
	Orr (ORI)	–	–	–	–	–	–	–	–	
	Prince of Wales (POW)*	no	Middle and Outer Southern	6	11	6	1.0000	0.0035	0.1150	
	Revillagigedio (REV)*	no	Inner Southern	5	10	5	1.0000	0.0040	0.0243	◊
	San Fernando (SNF)*	no	Middle and Outer Southern	3	1	3	1.0000	0.0009	0.5090	◊
	Suemez (SMZ)*	yes	Middle and Outer Southern	3	3	2	0.6670	0.0018	0.3070	
	Sukkwan (SWN)	–	–	–	–	–	–	–	–	
	Tuxekan (TXN)	no	–	–	–	–	–	–	–	
	Warren (WRN)	yes	Middle and Outer Southern	5	3	4	0.9000	0.0011	0.4480	
	Wrangell (WRG)*	no	Middle and Outer Southern	–	–	–	–	–	–	
	Zarembo (ZRB)*	no	Middle and Outer Southern	4	6	3	0.8330	0.0027	0.2320	
British Columbian Islands	Bischof (BSF)	–	–	–	–	–	–	–	–	
	Graham (GRM)	–	–	–	–	–	–	–	–	
	Hotsprings (HTS)	–	–	–	–	–	–	–	–	
	Moresby (MRB)	–	–	–	–	–	–	–	–	
	Ramsay (RMS)	–	–	–	–	–	–	–	–	
	Vancouver (VCR)	–	–	–	–	–	–	–	–	
Mainland	British Columbia (BC)	no	–	2	8	2	1.0000	0.0073	0.0000	◊
	Mainland Southeast Alaska (MLSE)	no	–	26	12	9	0.6650	0.0014	0.0000	
	Northern mainalnd Southeast (NMLSE)	no	–	–	–	–	–	–	–	
	Interior Alaska, Yukon and White Pass (North)	no	–	–	–	–	–	–	–	
	Cleveland Peninsula (CP/MLCP)*	–	Inner Southern	–	–	–	–	–	–	
	Foggy Bay (FB/MLFB)*	–	Middle and Outer Southern	–	–	–	–	–	–	◊
	Glacier Bay (GB/MLGB)*	–	Outer Northern	–	–	–	–	–	–	◊
	Haines (HNS/MLHNS)*	–	–	–	–	–	–	–	–	◊
	Interior Alaska (IAK/MLS)	–	–	–	–	–	–	–	–	
	Juneau (JNO/MLJNO)*	–	Inner Northern	–	–	–	–	–	–	◊
	Klukwan (KLU/MLKLU)	–	–	–	–	–	–	–	–	
	Misty Fjords (MFD/MLMFD)*	–	Inner Southern	–	–	–	–	–	–	◊
	Southeast Central (SEC AK/MLSEC)*	–	–	–	–	–	–	–	–	
	Southeast North (SEN AK/MLSEN)	–	–	–	–	–	–	–	–	
	Southeast South (SES AK/MLSES)	–	–	–	–	–	–	–	–	
	Skagway (SKW/MLSKW)	–	–	–	–	–	–	–	–	
	Taiya River (TYR/MLTYR)	–	–	–	–	–	–	–	–	
	White Pass (WP/MLWP)	–	–	–	–	–	–	–	–	
	British Columbia ‐ Central (BCC)*	–	–	–	–	–	–	–	–	
	British Columbia ‐ North (BCN)	–	–	–	–	–	–	–	–	
	British Columbia ‐ South (BCS)	–	–	–	–	–	–	–	–	
	Washington (WA)	–	–	–	–	–	–	–	–	
	Yukon Territory ‐ Central (YTC)	–	–	–	–	–	–	–	–	◊
	Yukon Territory ‐ South (YTS)*	–	–	–	–	–	–	–	–	◊

Note

Refugia = potential refugial islands and hypothesized island groups = island and adjacent mainland populations based on paleoshoreline reconstruction and LGM glacial cover = not included in the analyses that require the given information. Localities included in the Shimodaira‐Hasegawa tests are indicated with asterisk. *n *= haploid sample size; *S *= variable sites; *h *= # haplotypes; *Hd *= haplotype diversity; *π *= nucleotide diversity; mean multilocus *F*_ST_; ◊ = distinctive cyt *b* lineages.

### 2.3 Phylogenetic and demographic analyses

Lineage history was inferred through the use of a multilocus coalescent approach (Carstens & Knowles, 2007; Edwards, Liu, & Pearl, 2007; Maddison, 1997) with *beast (Heled & Drummond, 2010) which uses a Bayesian Markov chain Monte Carlo (MCMC) algorithm implemented in beast. All phased mtDNA and nuDNA loci were tested for molecular clock suitability and assigned as independent and unlinked and set with substitution models calculated in modeltest (Supporting Information Appendices S3 and S4). A priori groupings were designated based on geographic populations (e.g., islands) with initial evaluation of cyt *b* data (Supporting Information Appendices S4 and S5). A lognormal relaxed clock was designated for Cyt *b* with the same rates as the beast analysis (Supplementary text), while all rates for phased nuclear loci were estimated and assigned strict clocks. Each run consisted of random start trees with a Species Tree: Yule process prior and piecewise linear and constant root population size model with MCMC chain lengths of two billion iterations, sampling every two million. tracer, awty, logcombiner, and treeannotator were used as in the beast analysis (Supporting Information). phylogeoviz (Tsai, 2011) was used to visualize phased nuclear haplotype frequencies across the landscape.

Net genetic distances among major clades of cyt *b* were calculated in mega. To test for recent demographic change, we computed mtDNA and nuDNA summary statistics (segregating sites [*S*], number of haplotypes [*h*], haplotype diversity [*Hd*], and nucleotide diversity [*π*]), and selection and expansion statistics Tajima's (1989) *D*, Fu's (1997) F*s*, and R_2_ (Ramos‐Onsins & Rozas, 2002) with 10 thousand coalescent simulations for each phased locus in dnasp 5.10.1 (Librado & Rozas, 2009). Selection was also assessed through an HKA Test (Hudson, Kreitman, & Aguade, 1987).

To identify signals of population fluctuation, we estimated historical demography for the Island clades (clade includes both island populations and nearby mainland, and are distinct from Northern or Southern continental mitochondrial phylogroups) within cyt *b*, we generated Bayesian Skyline Plots implemented in beast. Three runs per analysis used a MCMC chain of two billion steps, sampled every two million, with strict molecular clocks and models of evolution (Supporting Information Appendix S4) calculated via modeltest. tracer and awty were used to assess convergence.

## 3 RESULTS

### 3.1 Identification of potential refugial locations

Predictive performance for SDMs had mean AUC values of 0.801 ± 0.067 for *M. longicaudus*, 0.777 ± 0.080 for *P. keeni,* and 0.754 ± 0.082 for *S. monticola* across replicate runs, and all performed significantly better than random. No model clamping was detected. Suitable climate conditions for all three species in Southeast Alaska were present across all four temporal periods (Figure 2), including in areas west of the glacial ice during the LGM. Greatest suitability was for *P. keeni* for all historic periods. Future distributions suggest a decrease in habitat suitability for the outer southern islands and increased suitability for mainland regions for all three species (Figure 2). Paleoshoreline reconstructions (Figure 3) at 10 kya suggest four major island groups (outer northern, inner northern, inner southern, and outer southern and middle islands) with potential northern and southern coastal refugia at the LGM. By 8 kya, contemporary island topography was present. Post‐glacial inter‐island colonization pathways from refugial locations were inferred from island connectivity (Figure 3).

### 3.2 Phylogenetic and demographic analyses

All loci across all species had varying levels of polymorphism and genetic diversity (Table 1 and Supporting Information Appendix S4), with mtDNA cyt *b* being the most variable locus. Among Island clades of all three species, *M. longicaudus* had the highest mtDNA haplotype diversity (98.4%), followed by *P. keeni* (97.8%), and *S. monticola* (76.4%). Nuclear haplotype diversity for *M. longicaudus* ranged from 12.4% to 18.2%, *P. keeni* 6.5%–39.1%*,* and *S. monticola* 4.1%–33.8%. Selection was not detected through HKA tests.

**Figure 4:**
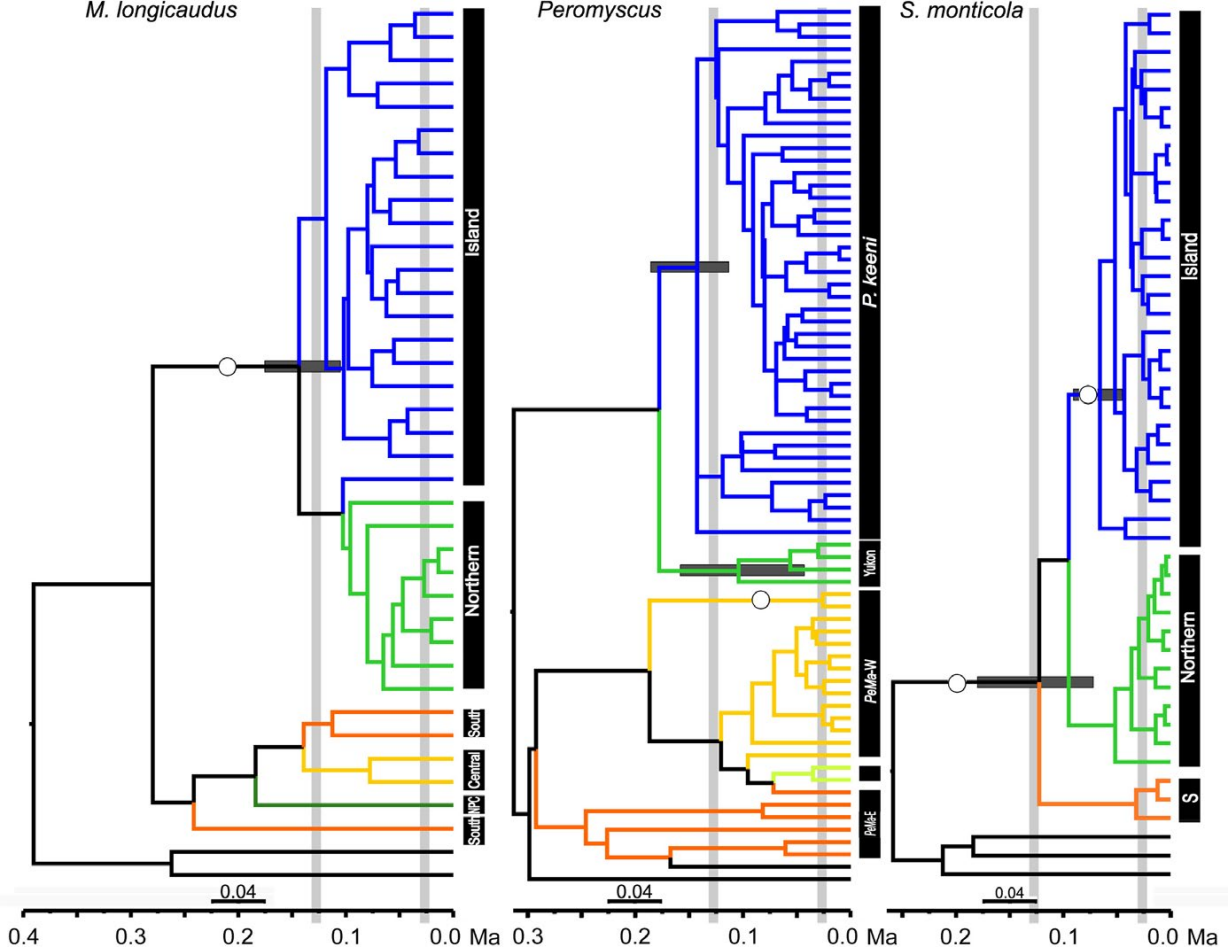
Multilocus Bayesian Species Tree. Posterior probabilities of ≥0.95 are represented with open circles on branches of the consensus tree. A priori groupings were designated based on cyt *b* Bayesian supported (≥0.95 posterior probability) clades. Blue = Island/*Peromyscus keeni*, bright green = Northern/*Peromyscus *sp. nov. (Yukon), dark green = North Pacific Coast, light yellow‐green = Colorado Plateau/*P. maniculatus* Southwest, golden = Central/*P. maniculatus* West, orange = Southern/*P. maniculatus* East, black = outgroups. Horizontal gray bars represent divergence date estimates and vertical bars indicate approximate time for the LIG and LGM. LGM: Last Glacial Maximum; LIG: Last Inter‐Glacial

The multilocus species trees (Figure 4) for *M. longicaudus* reveal a single supported clade containing both Island and Northern cyt *b* clades. The species tree for *Peromyscus *lacked support for the Island clade (*P. keeni*). Species tree for *S. monticola *supports the Island clade and the Southern clade, and also indicates that *S. monticola* is monophyletic.

Nuclear haplotypes within the AA are broadly distributed across the archipelago and exhibit little geographic structure for all loci and all species, with the exception of ETS2 in voles. *Microtus longicaudus* populations on Forrester and Chichagof Island each have unique haplotypes for ETS2 (Supporting Information Appendices S6 and S7). Multilocus divergence dates (Table 2) for the Island‐Northern clade of *M. longicaudus* and Island clades of *P. keeni* are near the LIG, while the Island clade for *S. monticola* diverged earlier, between the LGM and LIG.

**Table 2:** Divergence date estimates for the island lineages of *Microtus longicaudus*, *Peromyscus keeni,* and *Sorex monticola* based on both cyt *b* and phased multilocus analysis

Species	Lineage	cyt*b*			Multilocus		
		95% HPD lower	Mean	95% HPD upper	95% HPD lower	Mean	95% HPD upper
*M. longicaudus*	Island	156,100	215,700	285,900			
	Northern/Island	296,500	402,000	516,400	108,900	143,500	179,300
*Peromyscus*	*P. keeni*	207,500	316,500	438,400	114,900	144,200	177,800
	*Peromyscus *sp. nov.	69,103	194,600	339,700	45,300	105,800	167,000
*S. monticola*		475,900	756,400	1,037,400	72,100	122,400	180,000
	Island	72,200	114,400	166,300	45,000	65,300	90,500
	Southern	49,900	130,200	219,800			

Within the AA, distinctive but minimally divergent, mitochondrial lineages are consistently recorded across the three species for Forrester, Noyes and Revillagigedo islands each, likely reflecting their contemporary isolation. Distinct island‐specific lineages were also identified for Coronation, Dall, Kuiu, Lulu, Prince of Wales, and Zarembo for both *M. longicaudus* and *P. keeni*, and San Fernando for both *P. keeni* and *S. monticola *(Table 1; Supporting Information Appendices S5 and S8). Distinctive island lineages were recovered for Kupreanof, Suemez and Wrangell for *M. longicaudus*; while Admiralty, Baranof, Chichagof, Gravina, Heceta, and Warren were distinctive for *P. keeni;* and only Etolin was distinctive for *S. monticola* alone. Within *P. keeni*, the presence of a lineage representing the northern islands of Admiralty, Baranof and Chichagof is consistent with a biogeographic subregion first proposed by Swarth (1936; but see MacDonald & Cook, 1996).

**Figure 5:**
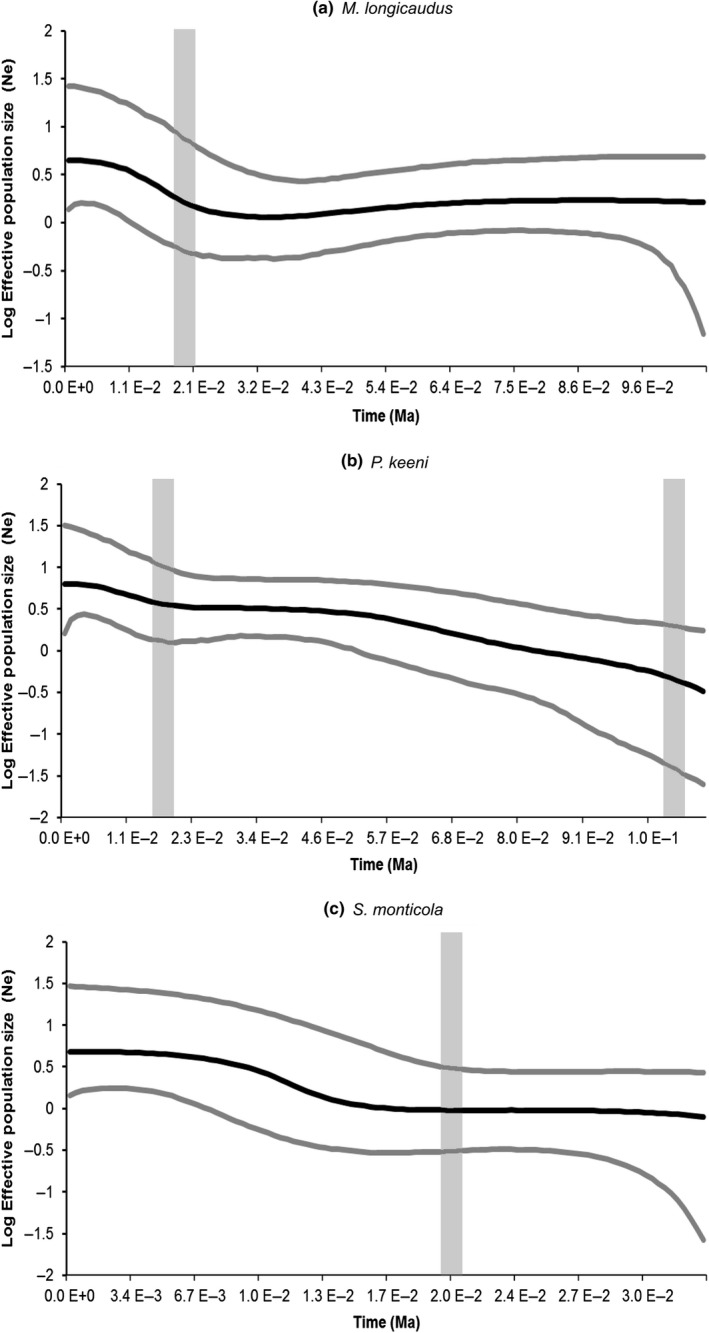
Cyt *b* Bayesian skyline plots (cyt*b* data only) for the major cyt*b* lineage populations: (a) *Microtus longicaudus* Island, (b) *Peromyscus keeni*, and (c) *Sorex monticola* Island. The *x*‐axis right‐to‐left from past (TMRCA) to present and is scaled in millions of years and the* y*‐axis is the log effective population size scaled by generation time. Vertical gray bars indicate the LIG (when applicable, right) and LGM (left) for reference. LGM: Last Glacial Maximum; LIG: Last Inter‐Glacial

Overall, measures of genetic diversity for the Island clades were low for all three species (Supporting Information Appendix S4), indicative of either population demographic expansions or selective sweeps. However, the inconsistent diversity indices among refugial versus nonrefugial islands could be a result of fixation (lower diversity) due to smaller island population size (Bidlack & Cook, 2002) compounded by a complex history of colonization and likely bottlenecked populations across the islands (Cook & MacDonald, 2013). Although significant expansion statistics can indicate selection, negative HKA tests suggest otherwise although we cannot rule out selection on closely linked regions. Additionally, significantly negative *D* and *F_S_* for all cyt *b* may be a result of recently expanded populations. The cyt *b* skyline plots (Figure 5) for *M. longicaudus* suggests increase in effective population size (N_e_) from a small ancestral population. In contrast to sudden growth in *M. longicaudus*, *P. keeni* and *S. monticola* show a steady increase in N_e_. Additionally, growth occurs post‐LGM within *S. monticola *and concurrent with the LGM for both *M. longicaudus *and *P. keeni. *Relative to mainland clades, high genetic diversity values for the Island clades of *M. longicaudus*, *P. keeni*, and *S. monticola* likely are the cumulative result of independent genetic drift within each island, with each of these fragmented island populations having relatively small N_e_.

### 3.3 Testing phylogenetic models under the Coastal Refugia Hypothesis

Differences in genetic divergence within and between areas identified as potential refugia by SDMs and those covered in ice were not detected (Supporting Information Appendix S9). Fu's *F_S_* and Tajima's *D* and diversity indices (Supporting Information Appendix S4) varied in significance. The Bayesian skyline plots (Supporting Information Appendix S10) for all populations of *M. longicaudus*, *P. keeni*, and *S. monticola* are unable to distinguish between refugial and colonized areas.

## 4 DISCUSSION

Phylogeographic studies help us understand how past environmental history has influenced genetic structure, but historical context (Grant & Grant, 2003) also provides a crucial foundation for forecasting how anthropogenic impacts, such as habitat conversion (e.g., old‐growth logging) or climate‐warming, will structure insular populations (Christensen et al., 2007; Fahrig, 2003; Olson, 1989). We found that genetic structure in three sympatric small mammals of the Alexander Archipelago was influenced by a complex history of deep isolation and subsequent colonization. Genetic footprints, combined with assessment of paleoecology, helps identify both past refugial locations and the contemporary geographic barriers that now structure populations. In the case of the AA, genetic structure in three co‐distributed species was influenced by ice cover and lower sea levels—factors that left paleoendemic signatures reflecting their longer‐term in situ divergence. Those isolates subsequently served as source populations for recolonization throughout the archipelago. A dominant feature in the data is an overall signal of island endemism and mainland demographic expansion, with idiosyncratic spatial (island) and temporal patterns among the three study species. Signals of overall demographic expansion among all three species across the entire AA and along the adjoining mainland are consistent with the Coastal Refugia Hypothesis, although details of histories differed across species.

### 4.1 Shared geologic and climatic history

Historic SDMs are consistent with the paleoendemic signatures and identify that suitable environmental conditions existed in Southeast Alaska for these species during both the LIG and LGM (Figure 2). Multilocus estimates of divergence for *M. longicaudus*, *P. keeni* and *S. monticola* suggest pre‐LGM initiation of regional divergence (Table 2). Coalescent simulations for both northwestern deer mice and dusky shrews are consistent with their long‐term persistence in the region, with both *P. keeni *and the Island clade of *S. monticola* distinctive. Additionally, minimal intraclade variability and inter‐island diversification characterized the Island clade of *S. monticola* (Figure 4 and Supporting Information Appendix S5). Coalescent simulations suggest a more recent divergence of *M. longicaudus*, potentially reflecting geographic proximity and relatively recent segregation (20 kyr) between contemporary populations representing the Island and Northern cyt b clades, rather than post‐glacial expansion of mainland populations into the AA (Sawyer & Cook, 2016). Inconsistent estimates within each species partially stem from our inability to calibrate trees with fossils, and thus account for rate decay (Ho, Phillips, Cooper, & Drummond, 2005). Other signals (i.e., diversity indices, Fu's F_s_; Tajima's D; Supporting Information Appendix S4) within *M. longicaudus* suggest a deeper history in Southeast Alaska, including their possible persistence and divergence in coastal refugia.

Demographic expansion was identified in the Island clades of all three species (Bayesian skyline plots and expansion statistics), consistent with deglaciation of these areas (Figure 5; Supporting Information Appendix S4). All three Island clades have lower estimates of mitochondrial and nuclear diversity, compared to their continental counterparts, perhaps reflecting the influence of historically small population sizes. When tested as a single population, rather than individual islands, coalescent simulations (i.e. expansion statistics; Supporting Information Appendix S4) identified the source populations as originating from the islands of Southeast Alaska, rather than mainland. The deeper history of Island clades and relatively higher numbers of endemics within this coastal region for each species (Cook & MacDonald, 2001) is most consistent with their extended persistence in the region followed by contemporary isolation across the fragmented archipelago, a finding that corresponds to the history of ermine in the region (Colella et al., 2018).

Overall, there are signals of shared history across *M. longicaudus*, *P. keeni*, and *S. monticola*, but the idiosyncratic influence of mutation rates, selection and drift, combined with independent population‐level responses to historical climate and variable pathways are potentially reflected in incongruent aspects of the phylogeographic patterns. Nuclear loci for all three species lack consistent signatures of geographic structure across the region (Figure 4 and Supporting Information Appendix S6 and Appendix S7), potentially due to a combination of incomplete lineage sorting and differential rates of male‐biased dispersal (Foster, 1965; Helmus, Mahler, & Losos, 2014; McCabe & Cowan, 1945). The possibility of human‐mediated transportation seems unlikely given island‐specific resolution of mtDNA. The mtDNA data are not consistent with our original prediction that these widespread species should have relatively high levels of gene flow across the region. Instead, the data suggest that repeated genetic exchange or admixture during periods of lowered sea levels throughout the late Pleistocene and early Holocene have been followed by segregation and divergence in these species.

### 4.2 Coastal Refugia Hypothesis

Although we are just beginning to explore the complex AA, preliminary studies suggest a significant role for northern coastal refugia in diversifying and structuring contemporary communities (e.g., de Volo et al., 2013; Hannon et al., 2010; Shafer et al., 2011). Reconstruction of paleoshorelines is complex due to non‐linear changes as a result in lithospheric rebound (Josenhans, Fedje, Pienitz, & Southon, 1997) and submerged signatures of glacial extent, but our reconstructions of historical island connectivity, and potential colonization pathways suggest the potential for multiple LGM glacial refugia in Southeast Alaska (Figures 2 and 3): (a) mainland near Glacier Bay, (b) outer Baranof and Chichagof islands, (c) Forrester refugial complex, which would possibly result in post‐glacial colonization through Prince of Wales, Zarembo and Mitkof islands, (d) Coronation refugial complex, colonization through Kuiu and Kupreanof islands, or (e) Annette‐Duke refugium, south of Gravina Island. Deep ocean trenches likely would have forced recolonization of Admiralty, Wrangell, and Etolin islands from populations on the mainland, rather than from direct island connections.

Species distribution models (Figure 2) for each species suggest suitable climate supported offshore habitat on the exposed shelf and select western islands (Table 1) since at least the LIG. Relatively high levels of infraspecific differentiation and timing of interclade divergence, coupled with their absence from Baranof Island (and Chichagof for *S. monticola*) indicate that *M. longicaudus* and *S. monticola* likely persisted in coastal refugia along the southern extent of the AA. In contrast, *P. keeni*, likely persisted in a combination of northern and southern refugia within the AA, as suggested by the distinct cyt *b *lineage for the northern islands and differentiation across the southern islands. Unique haplotypes for *P. keeni *individuals from the islands of Haida Gwaii also present the option of coastal refugia near Haida Gwaii (Hetherington et al., 2003).

Cowan (1935) suggested both *P. keeni* and *S. monticola* survived the Wisconsinan glaciation in coastal refugia in the AA. Although Klein (1965) and others (Heaton & Grady, 2003, 2007) conclude all small mammals likely failed to survive the LGM, there are pre‐LGM fossils of long‐tailed voles from Prince of Wales Island (Heaton & Grady, 2003, 2007). Lack of fossils on Prince of Wales during the LGM does not eliminate the possibility, however, that these species persisted further west in coastal refugia on the continental shelf when oceans were >120 m lower during the LGM.

Presence of paleoendemic lineages has implications for the application of island biogeographic theory to the AA (e.g., Conroy et al., 1999), early human colonization of the New World (Achilli, Olivieri, Semino, & Torroni, 2018), and for understanding the evolution of continental biota (Riddle, 2016). Island isolation in particular may require special consideration as measurements of isolation generally assume the source population is on the mainland. If source populations for some species were actually from coastal refugia, then diversity measurements would be complicated due to multiple colonization sources and routes (Figure 3), a conclusion consistent with Lucid and Cook (2004) who showed that island area had more influence on contemporary island genetic diversity, than isolation (as measured by distance from the mainland). For example, the source for Prince of Wales populations would traditionally be measured from the mainland immediately to the east, but source populations might instead be from refugia to the west (e.g., Forrester or Coronation complexes).

Diversification of fauna in coastal refugia and then subsequently on the Alexander Archipelago also raises the possibility that continental diversity in northwestern North America has been influenced through recolonization of the mainland from islands (e.g., Filardi & Moyle, 2005). Each of the small mammals in this cohort appears to have a distinctive clade present in the AA which does not extend far beyond SE AK. The range of the Island clade of *M. longicaudus* is limited to the AA and nearby mainland, a distribution consistent with their paleoendemism in the region (Figure 1). The distributions in both *P. keeni* and the Island clade of *S. monticola* (south to Washington) extend beyond SE Alaska, but divergence dates, net genetic distance, genetic diversity, and expansion statistics, as well as models of refugial migration for *P. keeni* (Sawyer, Flamme, Jung, MacDonald, & Cook, 2017), suggest these species persisted along the coast during the LGM. The finding of an island paleoendemic clade for all three species is consistent with the phylogeographic pattern uncovered for ermine (*Mustela erminea*) in the region (Colella et al., 2018), but differs somewhat from two other carnivores, Pacific Coast marten (*Martes caurina*) and black bear (*Ursus americanus*), where the distribution of the island lineages now extend far beyond the boundaries SE AK (Dawson et al., 2014; Fleming & Cook, 2002; Peacock et al., 2007; Small, Stone, & Cook, 2003).

## 5 CONCLUSION

Historical climate and coastal refugia shaped genetic structure of species of the high‐latitude Alexander Archipelago. Multiple lines of evidence suggest all three small mammals have paleoendemic lineages in the region, a finding consistent with other recent work on endemics in the region such as the Prince of Wales/Haida Gwaii ermine (Colella et al., 2018). Although this broad spatial pattern is concordant, questions remain regarding whether the timing of divergence coincides across these taxa. Cyclic climatic changes may produce similar spatial patterns that have different temporal signatures. Signals of demographic expansion across the region for these distinctive clades are also evident and roughly concordant. More detailed documentation of Late Quaternary changes in sea level and glacial cover along the North Pacific Coast, in addition to expanded genome‐scale sampling of these and other endemic organisms, however, are needed to refine the number, location, and influence of glacial refugia.

Assessments of genetic structure across an array of species in complex landscapes, such as this coastal archipelago which experienced dynamic sea level fluctuations (e.g., Dolby et al., 2018), provide an initial framework for scientifically defensible management decisions (Gutrich et al., 2005; Pritchard, Jones, & Cowley, 2007). Future SDMs for these species forecast serious impacts, especially on the outer islands of the AA (Figure 2). Those outer islands now support a disproportionate number of subspecies (Cook & MacDonald, 2001) and endemic lineages of mammals (Cook et al., 2006) and other taxa (Sikes & Stockbridge, 2013). Those islands also have experienced extensive anthropogenic habitat conversion (e.g., clear‐cut logging of old‐growth forests) over the past six decades with only minimal monitoring of impacts on biodiversity (Cook et al., 2006; Orians & Schoen, 2013). More generally, similarities across species are identifiable through the use of integrated analyses (i.e., phylogenetic reconstructions, SDMs); approaches that could be extended to other taxa in the AA or other high‐latitude fragmented systems, such as Haida Gwaii of British Columbia (Reimchen & Byun, 2006), the Japanese Archipelago (Millien‐Parra & Jaeger, 1999), or British Isles (Vincent, 1990) to help conserve regionally distinctive biota experiencing dynamic environmental change (Avise, 2008; Hendry et al., 2010).

## CONFLICT OF INTEREST

None declared.

## AUTHOR CONTRIBUTION

This research was part of the doctoral dissertation of Y.E.S. She is interested in the effects of historical climate on contemporary small mammals, particularly in the high latitude islands of Southeast Alaska. Y.E.S and J.A.C. developed the ideas; S.O.M. and J.A.C collected the specimens; Y.E.S. collected the genetic data in the laboratory of J.A.C; Y.E.S. analyzed the data; and Y.E.S., S.O.M., E.P.L. and J.A.C contributed to the writing.

## Data Availability

Sample locations with museum numbers, latitude and longitude, major cyt *b* clade, and associated GenBank accession numbers (Supporting Information Appendix S1), as well as between group net genetic divergences of cyt b among refugial and nonrefugial Southeast Alaskan populations lineages of *M. longicaudus*, *P. keeni* and *S. monticola* (Supporting Information Appendix S9) are available from the Dryad Digital Repository: https://doi.org/10.5061/dryad.867g4c8.
