# Supplementary material for: Living on the edge: Exploring the role of coastal refugia in the Alexander Archipelago of Alaska
Source: Ecol Evol. 2019 Feb 1;9(4):1777–97. doi: 10.1002/ece3.4861 (PMC6392352; doi:10.1002/ece3.4861)

**Appendix I**

Specimens examined. Museum number acronyms are MSB= Museum of Southwestern Biology, UAM=University of Alaska Museum of the North, Fairbanks, HG= Gwaii Haanas National Park Reserve and Haida Heritage Site, and UWBM=University of Washington Burke Museum. GenBank numbers correspond to cyt *b*, and each phased allele for *M. longicaudus* (ETS2, FGB and Rag1), *Peromyscus* (FGB, IRBP and Zp3) and *S. monticola* (ADH2, ApoB and FGB) respectively, –= not applicable. GenBank in bold were obtained from other studies.

Data available from the Dryad Digital Repository: https://doi.org/10.5061/dryad.867g4c8

**Appendix II**

Primer list and PCR annealing temperatures. Primers used for amplification and sequencing mtDNA Cytochrome B (cyt*b*), and nuclear loci Alcohol Dehydrogenase 2 (ADH2), Apolipoprotein B (ApoB), Protein C-est-2 (ETS2), β-fibrinogen (FGB), interphotoreceptor retinoid-binding protein (IRBP), Recombination Activating Protein 1 (Rag1) and zona pellucida 3 (ZP3) for species indicated, including outgroups in each genus, with annealing temperatures (^o^C) indicated in parentheses.

Reaction mixtures of 1µl DNA extract, 1 µl of primer each (2mM), 1.5 µl PCR buffer (10x), 1.5 µl MgCl2 (25mM), 1.25 µl of dNTP’s (10mM ), 1.25 µl of Bovine Serum Albumen (BSA, 1.5mM), and 0.08 µl of AmpliTaq DNA polymerase (Applied Biosystems, Foster City, CA, USA) and were adjusted to a final volume of 15 µl with ddH2O. After cleaning PCR products with ExoSap-IT (Affymetrix, Santa Clara, CA), automated sequencing was conducted at either the High Throughput Genomic Center (Seattle, WA, USA) or using an Applied Biosystems 3110 DNA sequencer (Molecular Biology Facility, UNM) using original PCR primers and BigDye v3.1 (Applied Biosystems) terminator reaction chemistry.

| **Species** | **Primer** | **Sequence (5'-3')** | **Reference** |
| --- | --- | --- | --- |
|  | **cyt*b* (50)** | |  |
| *M. longicaudus* | L14724 |  | (Irwin *et al.* 1991; Kocher & White 1989) |
|  | Vole 14 |  | (Conroy & Cook 1999) |
| *Peromyscus &  S. monticola* | L14734 |  | (Ohdachi et al. 2001) |
|  | CytBRev |  | (Anderson and Yates 2000) |
|  |  |  |  |
| *S. monticola* | **ADH2 (50)** | |  |
|  | ADH2F |  | (Lyons *et al.* 1997) |
|  | ADH2R |  | (Lyons *et al.* 1997) |
|  |  |  |  |
| *S. monticola* | **ApoB (54.6)** | |  |
|  | ApoBF |  | (Dubey *et al.* 2007) |
|  | ApoBR |  | (Dubey *et al.* 2007) |
|  |  |  |  |
| *M. longicaudus* | **ETS2 (63)** | |  |
|  | ETS2F |  | (Lyons *et al.* 1997) |
|  | ETS2R |  | (Lyons *et al.* 1997) |
|  |  |  |  |
| *M. longicaudus* | **FGB (65)** | |  |
|  | MSB_MFGBF | CGTTTGGATTGGCGGAGTGG | This study, modified from Matocq *et al*. (2007) |
|  | MSB_MFGBR | GCACGTACGACAGGGACAACG | This study, modified from Matocq *et al*. (2007) |
|  |  |  |  |
| *Peromyscus* | **FGB (63)** | |  |
|  | MSB_PFGBF | GCCGTTTGGATTGGCTGC | This study, modified from Matocq *et al*. (2007) |
|  | MSB_PFGBR | CGACAGGGACAATGATGGC | This study, modified from Matocq *et al*. (2007) |
|  |  |  |  |
| *S. monticola* | **FGB (63)** | |  |
|  | MSB_SFGBF | GCCATCCTCTTTAGAACACTG | This study, modified from Matocq *et al*. (2007) |
|  | MSB_SFGBR | CGATGGCTGGTAGGCGTCC | This study, modified from Matocq *et al*. (2007) |
|  |  |  |  |
| *Peromysucs* | **IRBP (60)** | |  |
|  | MSB_PIRBPF | CCAGGAGGTACTGAGTGAGC | This study, modified from Stanhope *et al.* (1992) |
|  | MSB_PIRBPR | GCTGAGTAGTCCATGCTAGC | This study, modified from Stanhope *et al.* (1992) |
|  |  |  |  |
| *M. longicaudus* | **Rag1 (60)** | |  |
|  | MSB_Rag1F | GCAGTCTCCTTTAGTTCCAGAC | This study, modified from Steppan *et al*.(2004) |
|  | MSB_Rag1R | CCAACAGGAACAACGTCAAGC | This study, modified from Steppan *et al*.(2004) |
|  |  |  |  |
| *Peromyscus* | **Zp3 (56)** | |  |
|  | Z36FA |  | (Turner and Hoekstra 2006) |
|  | Z37RA |  | (Turner and Hoekstra 2006) |

Anderson, S. and T. L. Yates. 2000. A new genus and species of phyllotine rodent from Bolivia. Journal of Mammalogy 81:18-36.

Conroy, C. J. and J. A. Cook. 1999. MtDNA evidence for preated pulses of speciation within Arvicoline and Murid rodents. Journal of Mammalian Evolution 6:221-245.

Dubey, S., N. Salamin, S. D. Ohdachi, P. Barriere, and P. Vogel. 2007. Molecular phylogenetics of shrews (Mammalia : Soricidae) reveal timing of transcontinental colonizations. Molecular Phylogenetics and Evolution 44:126-137.

Irwin, D. M., T. D. Kocher, and A. C. Wilson. 1991. Evolution of the Cytochrome *b* gene of mammals. Journal of Molecular Evolution 32:128-144.

Kocher, T. D. and T. J. White. 1989. Evolutionary analysis via PCR. Stockton Press, New York.

Lyons, L. A., T. F. Laughlin, N. G. Copeland, N. A. Jenkins, J. E. Womack, and S. J. Obrien. 1997. Comparative anchor tagged sequences (CATS) for integrative mapping of mammalian genomes. Nature Genetics 15:47-56.

Matocq, M. D., Q. R. Shurtliff, and C. R. Feldman. 2007. Phylogenetics of the woodrat genus *Neotoma* (Rodentia: Muridae): Implications for the evolution of phenotypic variation in male external genitalia. Molecular Phylogenetics and Evolution 42:637-652.

Ohdachi, S., N. E. Dokuchaev, M. Hasegawa, and R. Masuda. 2001. Intraspecific phylogeny and geographical variation of six species of northeastern Asiatic *Sorex* shrews based on the mitochondrial cytochrome *b* sequences. Molecular Ecology 10:2199-2213.

Stanhope, M. J., J. Czelusniak, J.-S. Si, J. Nickerson, and M. Goodman. 1992. A molecular perspective on mammalian evolution from the gene encoding interphotoreceptor retinoid binding protein, with convincing evidence for bat monophyly. Molecular Phylogenetics and Evolution 1:148-160.

Steppan, S. J., R. M. Adkins, and J. Anderson. 2004. Phylogeny and divergence-date estimates of rapid radiations in muroid rodents based on multiple nuclear genes. Systematic Biology 53:533-553.

Turner, L. M. and H. E. Hoekstra. 2006. Adaptive evolution of fertilization proteins within a genus: Variation in ZP2 and ZP3 in deer mice (*Peromyscus*). Molecular Biology and Evolution 23:1656-1669

**Appendix III**

**Materials and Methods**

*Phylogenetic and demographic analyses*

To explore the phylogenetic relationship within each species, we performed Maximum Likelihood (ML) and Bayesian phylogenetic reconstructions for cyt *b* for each species. We used ModelTest (Posada and Crandall 1998; Posada and Buckley 2004) to determine genetic models of evolution for each locus (Table 1). ML estimations were performed in mega with 1000 bootstrap replicates. Using Beast v1.7.5 (Drummond et al. 2012), we generated Bayesian phylogenies and divergence dates estimates with input files prepared in BEAUti v1.7.5., part of the Beast software package. Because we lacked reliable fossils, we were unable to incorporate fossil calibration into either cyt *b* or species trees. All loci were tested for molecular clock suitability and set to strict molecular clocks with rates for phased nuclear loci estimated based on previous cyt *b* estimates. An *a priori* uncorrelated lognormal relaxed clock was set for cyt *b* with a mutation rate of 4% Myr^-1^ assigned to *M. longicaudus* and *P. keeni* (Conroy and Cook 1999; discussed in Brunhoff et al. 2003 as divergence rate of 6 - 10% per Myr; Hope et al. 2013 as mutation rate of 4% per Myr) and a rate of 5.5% Myr^-1^ for *S. monticola* (Hope et al. 2013). A Bayesian uncorrelated relaxed clock was used to reduce errors associated with recent divergence times and the lack of reliable calibration points (Drummond et al. 2006; Ho and Duchene 2014). We applied a coalescent constant size tree prior with a random start tree. We implemented an uncorrelated lognormal relaxed clock because relationships with non-insular populations are older, for 60 million generations, and sampled every 2000. Time to Most Recent Common Ancestor (TMRCA) was determined with a 95% PP distribution in Tracer v1.5 (Rambaut and Drummond 2007). For each tree, convergence statistics were assessed with both a minimum effective sample size (ESS) value of 200 and trace graphs in Tracer with convergence onto the same tree analyzed with the online software awty (Nylander et al. 2008). Three independent runs were combined using LogCombiner v1.7.5, with a 10% burn-in and tree files were annotated in TreeAnnotator v1.7.5 (part of Beast software package). Tree topology was visualized in FigTree v1.4.0 (Rambaut 2009), with supported clades designated with a 95% support for Bayesian and 70 for ML.

**Results**

*Phylogenetic and demographic analyses*

Cyt *b* phylogenies identify independent Island clades for all three species. The Island clade of the vole, *M. longicaudus,* is restricted to Southeast Alaska, southern Yukon, and south-central Alaska (Appx. V) and contains 16 lineages (identified by a ≥0.95 posterior probability). This species is notably absent from Baranof Island in the AA as well as Haida Gwaii and Vancouver Island to the south. Representatives of the Island and Northern clades contact in northern Southeast Alaska near Haines and Juneau and are in close geographic proximity farther south along the mainland coast.

For deermice, *P. keeni* represents the Island clade (Supplementary Figure 1). *Peromyscus keeni* ranges from southern Yukon through Southeast Alaska and coastal British Columbia to Washington’s Olympic Peninsula (Figure 7) and contains substantial structure (25 lineages). There is contact between *P. keeni* and *P. maniculatus* in British Columbia and northern Washington along the east side of the Coast Mountains. These species are also in close proximity (Sawyer et al. *submitted*) in southern Yukon.

The Island clade of the shrew, *S. monticola,* is highly structured (10 lineages), ranging from Southeast Alaska (with the exception of Baranof and Chichagof islands) and eastern British Columbia southward into Washington, with possible contact with the Northern clade near Haines and Juneau, Alaska and farther south in Washington (Figure 7).

Within *M. longicaudus*, the Island clade is sister to the North Pacific Coast (NPC) and Northern clades at 1.2 ± 0.3% net genetic distance, and more highly diverged from the Southern clade (3.7 ± 0.5%; Appx. IX). For *P. keeni,* the Southwest clade of *P. maniculatus* (2.3 ± 0.4%) is closest to the island clade, *P. keeni*, followed by the Yukon *Peromyscus* sp. (3.6 ± 0.5%), and then other *P. maniculatus* (3.8 ± 0.5%). The Island clade of *S. monticola* is least diverged from the Northern clade (4.8 ± 0.5%), and most diverged from the Southern clade (5.4 ± 0.6%).

**Appendix IV**

Cyt *b* and nuclear diversity indices, expansion statistics and models of evolution. n=haploid sample size; L=length of sequence; S=variable sites; Eta=#mutations; h=#haplotypes; Hd=haplotype diversity; π=nucleotide diversity; D=Tajima's D; Fs=Fu's FS; r=raggedness index; R2=Ramos-Osnin's R2; Model=model of evolution as selected by ModelTest; ◊ = distinctive cyt *b* lineages. Bold values are significant at p<0.05 (p<0.02 for FS). Refugia = refugial islands, Non-refugia = non refugial islands (see Table 2).

| **Species** | **Group** | **Gene** | **n** | **L** | **S** | **h** | **Hd** | **π** | **D** | **Fs (p<.02)** | **R2** | **Model of Evolution** |
| --- | --- | --- | --- | --- | --- | --- | --- | --- | --- | --- | --- | --- |
| *M. longicaudus* | All Samples | Cytb | 140 | 1143 | 174 | 100 | 0.992 | 0.01423 | **-0.08804** | **-0.61525** | **0.08687** | TrN+I+G |
|  |  | ETS2 | 63 | 733 | - | - | - | - | - | - | - | GTR+I+G |
|  |  | FGB | 79 | 600 | - | - | - | - | - | - | - | HKY+G |
|  |  | Rag1 | 70 | 963 | - | - | - | - | - | - | - | HKY |
|  | No Outgroups | Cytb | 136 | 1143 | 128 | 96 | 0.992 | 0.01037 | **-0.09456** | **-0.57918** | **0.08725** | - |
|  |  | ETS2 | 61 | 733 | 14 | 14 | 0.477 | 0.002 | -0.04807 | **-0.1418** | **0.09083** | - |
|  |  | FGB | 77 | 600 | 18 | 19 | 0.371 | 0.00094 | **-0.00791** | **-0.18466** | **0.09204** | - |
|  |  | Rag1 | 68 | 963 | 9 | 10 | 0.182 | 0.00023 | **-0.00041** | **-0.10372** | **0.09885** | - |
|  | Island | Cytb | 86 | 1143 | 73 | 57 | 0.984 | 0.00519 | **-0.09551** | **-0.29755** | **0.09479** | HKY+I+G |
|  |  | ETS2 | 35 | 733 | 9 | 5 | 0.138 | 0.00061 | **-0.0251** | 0.00678 | **0.11424** | HKY+I+G |
|  |  | FGB | 42 | 600 | 7 | 8 | 0.182 | 0.00033 | **-0.0096** | **-0.01618** | **0.11433** | HKY+I |
|  |  | Rag1 | 39 | 963 | 3 | 4 | 0.124 | 0.00013 | **-0.00142** | **-0.00572** | **0.11697** | TrN |
|  | Refugia | Cytb | 36 | 1143 | 34 | 19 | 0.948 | 0.0052 | -0.10885 | -0.10906 | **0.1141** | HKY+G |
|  |  | ETS2 | 24 | 733 | 2 | 3 | 0.163 | 0.00024 | **-0.01231** | **0.20236** | **0.1708** | HKY |
|  |  | FGB | 30 | 600 | 4 | 3 | 0.297 | 0.00132 | -0.02833 | 0.08965 | **0.13912** | HKY |
|  |  | Rag1 | 28 | 963 | 2 | 3 | 0.489 | 0.00055 | -0.02278 | 0.14512 | **0.14835** | HKY |
|  | Non-refugia | Cytb | 50 | 1143 | 54 | 40 | 0.986 | 0.00555 | **-0.09545** | **-0.20269** | **0.10568** | TrN+I |
|  |  | ETS2 | 46 | 733 | 11 | 3 | 0.127 | 0.00123 | **-0.03223** | 0.04974 | **0.12001** | HKY |
|  |  | FGB | 54 | 600 | 6 | 7 | 0.244 | 0.00044 | **-0.00255** | **0.07172** | **0.12803** | TrN+I |
|  |  | Rag1 | 50 | 963 | 2 | 3 | 0.117 | 0.00012 | **-0.00293** | 0.09541 | **0.13511** | TrN |
| *Peromyscus* | All Samples | Cytb | 192 | 1143 | 209 | 107 | 0.984 | 0.01224 | **-0.09268** | **-0.69546** | **0.08185** | GTR+I+G |
|  |  | FGB | 109 | 479 | 26 | 19 | 0.24 | 0.00098 | **-0..02871** | **-0.29886** | **0.08599** | HKY |
|  |  | IRBP | 99 | 421 | 9 | 9 | 0.381 | 0.00116 | **-0.00929** | **-0.24871** | **0.08744** | HKY |
|  |  | Zp3 | 112 | 314 | 11 | 14 | 0.233 | 0.00094 | **-0.01977** | **-0.25383** | **0.08864** | HKY+I |
|  | No Outgroups | Cytb | 190 | 1143 | 145 | 109 | 0.984 | 0.01042 | **-0.07315** | **-0.55263** | **0.08163** | - |
|  |  | FGB | 107 | 479 | 21 | 18 | 0.228 | 0.00078 | -0.01356 | **-0.22712** | **0.08829** | - |
|  |  | IRBP | 96 | 421 | 9 | 9 | 0.391 | 0.0012 | **-0.03044** | **-0.21412** | **0.08876** | - |
|  |  | Zp3 | 109 | 314 | 9 | 12 | 0.223 | 0.00087 | **-0.015** | **-0.24393** | **0.08962** | - |
| *P. keeni* | Island | Cytb | 155 | 1143 | 89 | 80 | 0.978 | 0.00459 | **-0.0772** | -0.31143 | **0.08483** | GTR+I+G |
|  |  | FGB | 75 | 479 | 2 | 3 | 0.065 | 0.00014 | -0.10294 | -0.40733 | **0.08532** | TrN+I |
|  |  | IRBP | 63 | 421 | 4 | 5 | 0.391 | 0.00107 | -0.02635 | -0.12602 | **0.09724** | HKY+I |
|  |  | Zp3 | 76 | 314 | 8 | 9 | 0.199 | 0.00089 | -0.01263 | **-0.17179** | **0.09829** | HKY+I |
|  | Refugia | Cytb | 47 | 1143 | 42 | 29 | 0.97 | 0.00463 | **-0.08343** | **-0.15405** | **0.4077** | HKY+I |
|  |  | FGB | 40 | 479 | 4 | 5 | 0.395 | 0.0009 | -0.01661 | 0.11807 | **0.13538** | TrN |
|  |  | IRBP | 32 | 421 | 4 | 5 | 0.571 | 0.00171 | -0.01988 | 0.11493 | **0.13758** | HKY |
|  |  | Zp3 | 42 | 314 | 6 | 4 | 0.603 | 0.0066 | -0.07205 | -0.0308 | **0.11389** | TrN+I |
|  | Non-refugia | Cytb | 108 | 1143 | 100 | 68 | 0.984 | 0.00613 | **-0.08062** | **-0.3738** | **0.09076** | GTR+I+G |
|  |  | FGB | 110 | 479 | 0 | 1 | 0 | 0 | - | - | **-** | HKY |
|  |  | IRBP | 94 | 421 | 4 | 7 | 0.665 | 0.00224 | -0.02728 | -0.09094 | **0.09908** | HKY+I |
|  |  | Zp3 | 110 | 314 | 8 | 9 | 0.254 | 0.00111 | **-0.0272** | **-0.08196** | **0.10334** | HKY+I |
| *S. monticola* | All Samples | Cytb | 158 | 1140 | 138 | 58 | 0.87 | 0.01483 | **-0.08163** | -0.67266 | **0.08461** | GTR+I+G |
|  |  | ADH2 | 77 | 281 | 2 | 3 | 0.076 | 0.00028 | -0.08555 | -0.41299 | **0.08496** | HKY |
|  |  | ApoB | 55 | 500 | 30 | 12 | 0.62 | 0.00611 | **-0.08992** | -0.1969 | **0.09157** | HKY |
|  |  | FGB | 75 | 589 | 13 | 9 | 0.117 | 0.00058 | **-0.01859** | **-0.12373** | **0.09643** | HKY+I+G |
|  | No Outgroups | Cytb | 148 | 1140 | 71 | 51 | 0.852 | 0.00905 | -0.10863 | **-0.50509** | **0.08594** | - |
|  |  | ADH2 | 72 | 281 | 2 | 3 | 0.055 | 0.00025 | -0.10054 | -0.3663 | **0.08607** | - |
|  |  | ApoB | 50 | 500 | 5 | 5 | 0.541 | 0.00317 | -0.05092 | -0.13439 | **0.09457** | - |
|  |  | FGB | 68 | 589 | 4 | 4 | 0.044 | 0.00011 | **-0.08883** | -0.37778 | **0.08677** | - |
|  | Island | Cytb | 115 | 1140 | 38 | 31 | 0.764 | 0.00151 | **-0.06231** | **-0.15173** | **0.09154** | HKY+I+G |
|  |  | ADH2 | 48 | 281 | 1 | 2 | 0.041 | 0.00015 | -0.07474 | -0.21871 | **0.09318** | HKY+I |
|  |  | ApoB | 26 | 500 | 5 | 4 | 0.338 | 0.00155 | -0.04454 | 0.02484 | **0.1175** | HKY |
|  |  | FGB | 46 | 589 | 5 | 5 | 0.146 | 0.00032 | **-0.00623** | **-0.03408** | **0.11045** | HKY+I |
|  | Refugia | Cytb | 34 | 1140 | 25 | 16 | 0.913 | 0.00341 | -0.08419 | **-0.02008** | **0.1172** | GTR+I |
|  |  | ADH2 | 30 | 281 | 0 | 1 | 0 | 0 | - | - | - | JC |
|  |  | ApoB | 18 | 500 | 2 | 3 | 0.392 | 0.00084 | -0.01306 | 0.22258 | **0.17967** | HKY |
|  |  | FGB | 30 | 589 | 3 | 4 | 0.251 | 0.00046 | **-0.01126** | **0.14274** | **0.15434** | HKY |
|  | Non-refugia | Cytb | 81 | 1140 | 35 | 26 | 0.751 | 0.00146 | **-0.04799** | **-0.09461** | 0.09909 | HKY+G |
|  |  | ADH2 | 66 | 281 | 1 | 2 | 0.6 | 0.00022 | -0.07799 | -0.33319 | **0.0999** | HKY+I |
|  |  | ApoB | 34 | 500 | 3 | 2 | 0.299 | 0.0018 | -0.03143 | 0.08012 | **0.13118** | HKY |
|  |  | FGB | 62 | 589 | 4 | 4 | 0.182 | 0.00041 | **-0.01448** | 0.02697 | **0.12298** | HKY+I |

**Appendix V**

Dated Bayesian cyt*b* trees for *M. longicaudus*, *P. keeni* and *S. monticola*. Posterior probability ≥0.95 represented with open circles and Maximum Likelihood bootstraps of ≥0.7 with asterisks are shown on branches. Vertical light gray bars represent the LIG (left) and LGM (right). Dark gray horizontal bars = 95% CI for TMRCA for the Island clade for each species. Geographic location (Table 2) for supported intralineage clades are immediately right taxon tips. Major lineage abbreviations are: COP=Colorado Plateau; NPC=North Pacific Coast, PeMa=*P. maniculatus*, E (East), W (West) and SW (Southwest); S=South.


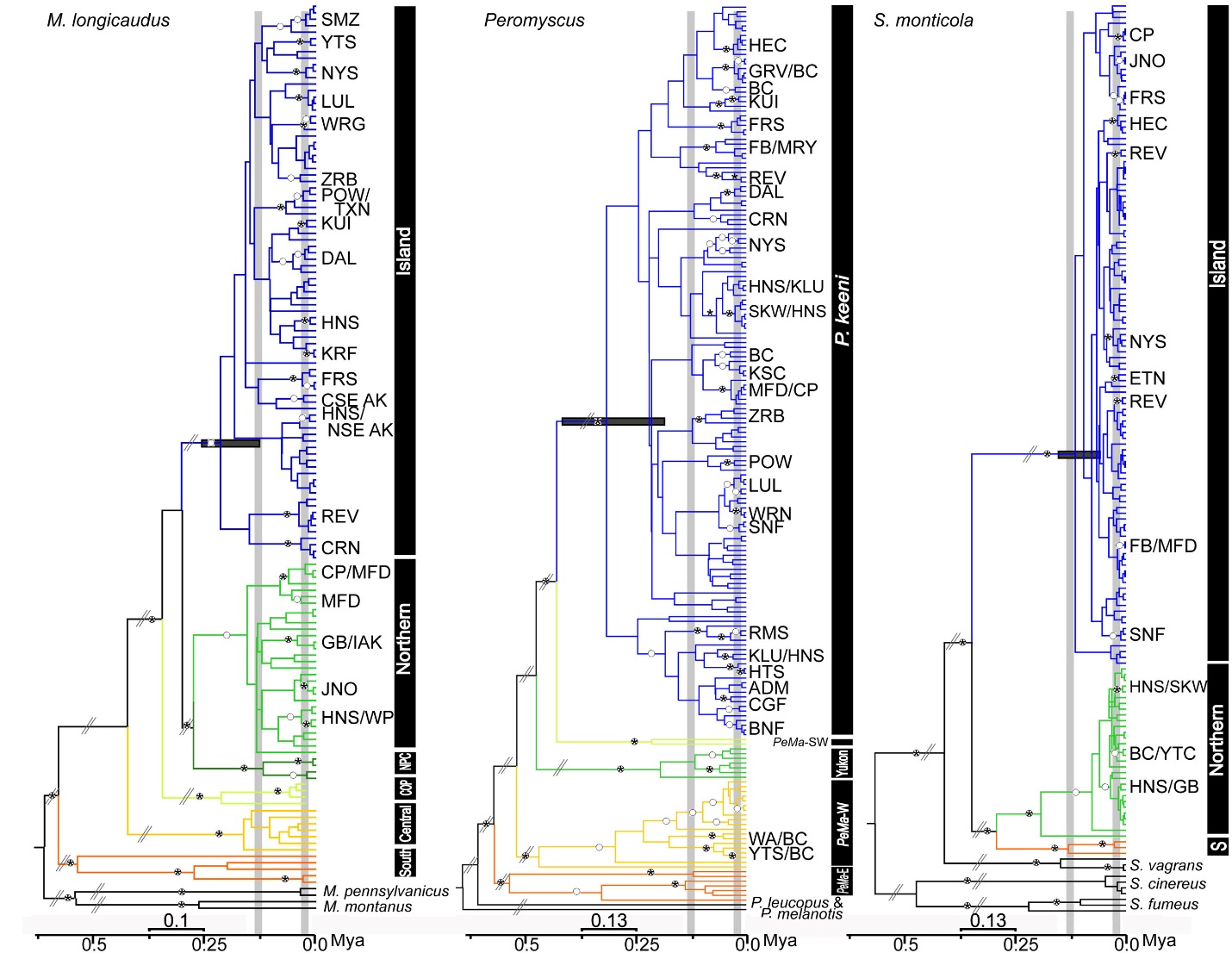


**Appendix VI**

Island phased nuclear haplotype distribution for *M. longicaudus*, *P. keeni* and *S. monticola* as prepared by Phylogeoviz.


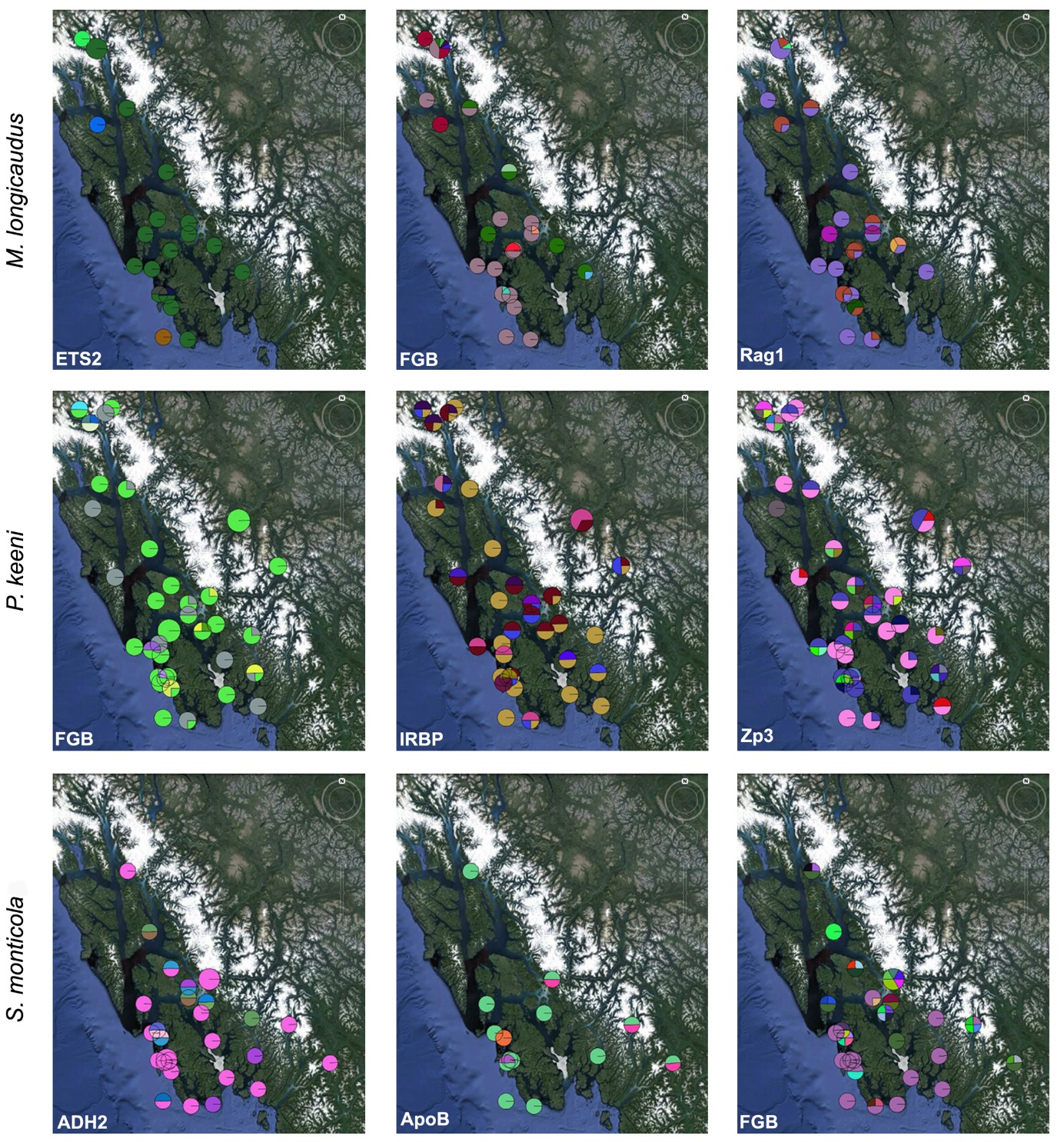


**Appendix VII**

Bayesian gene trees for phased nuclear loci for *M. longicaudus* (a. ETS2, b. FGB, and c. Rag1), *Peromyscus* (d. FGB, e. IRBP, and f. Zp3) and *S. monticola* (g. ADH2, h. ApoB, and i. FGB) with posterior probabilities of ≥0.95 represented with open circles on branches. Geographic locations for supported intralineage clades are provided. Blue = Island/*P. keeni*, bright green = Northern/*Peromyscus* sp. (Yukon), dark green = North Pacific Coast, light yellow-green = Colorado Plateau/ *P. maniculatus* Southwest, golden = Central/*P. maniculatus* West, orange = Southern/*P. maniculatus* East, black = outgroups.
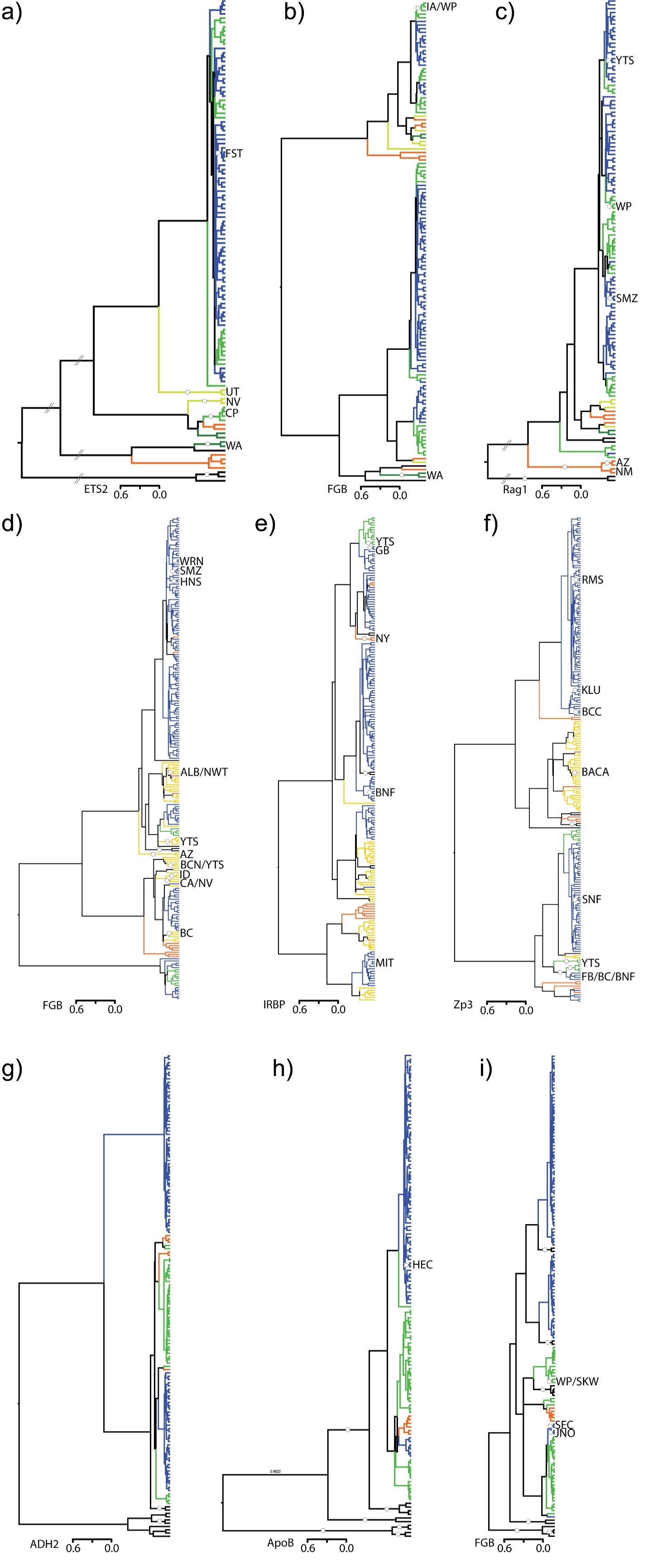


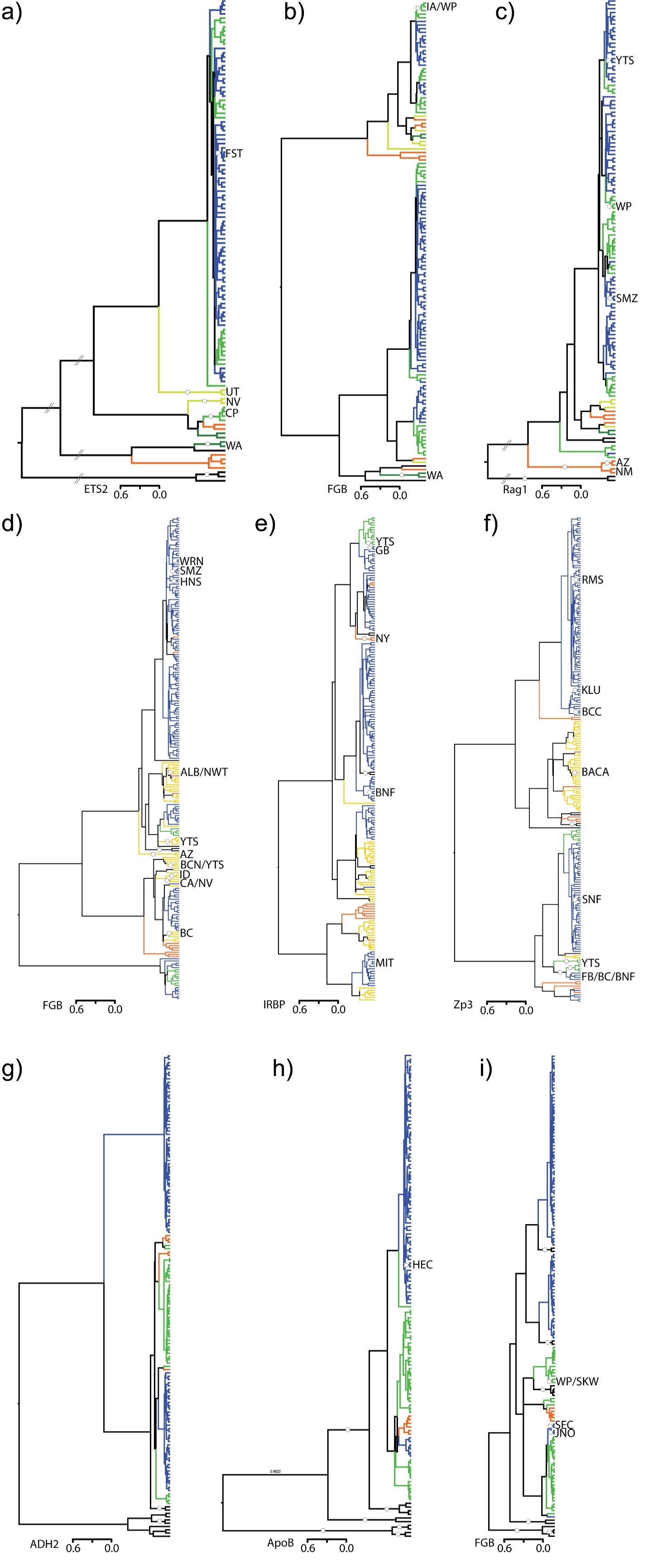

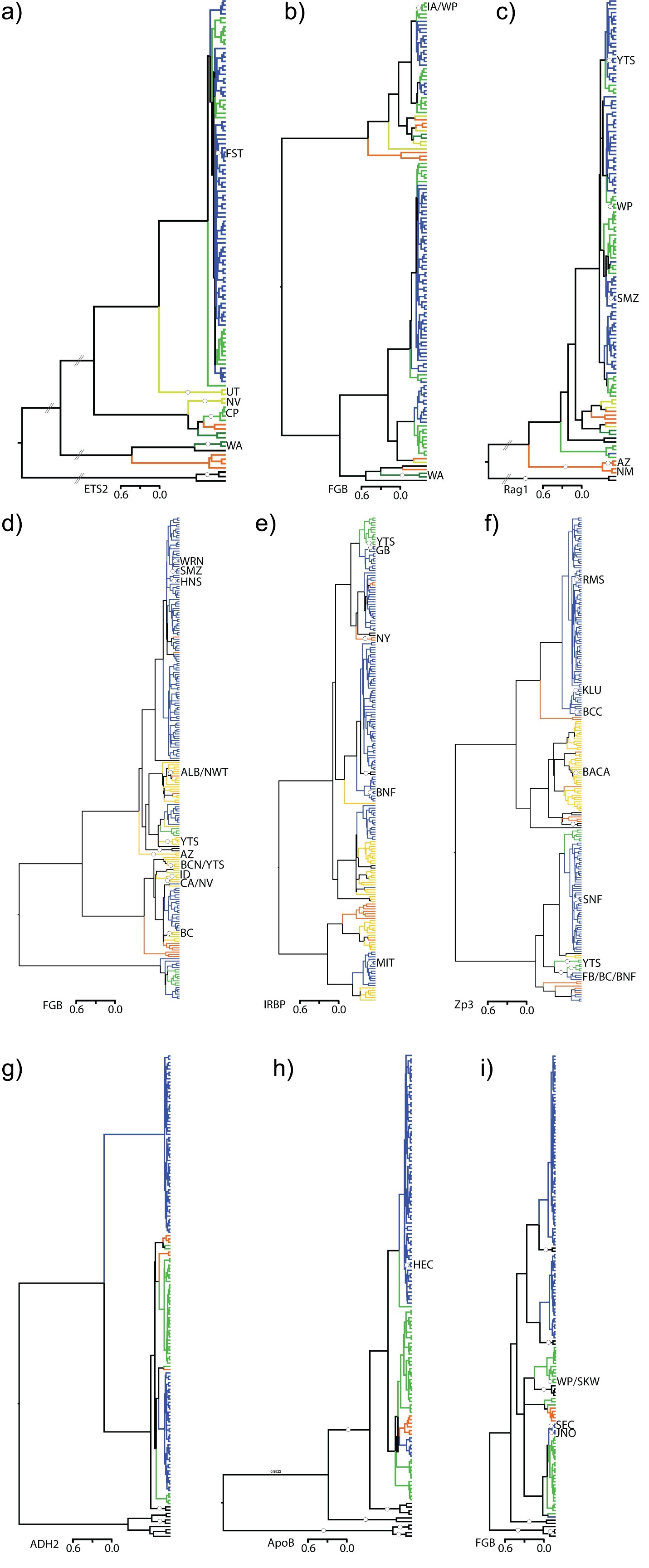


**Appendix VIII**

Between group net genetic divergences among major cyt *b* lineages of *M. longicaudus*, *Peromyscus* and *S. monticola*. The number of base differences per site from estimation of net average between groups of sequences is shown. Standard error estimate(s) are shown above the diagonal. All ambiguous positions were removed for each sequence pair. Evolutionary analyses were conducted in mega5.

| *M. longicaudus* | Central | Colorado Plateau | Island | Northern | North Pacific Coast | Southern | *M. pennsylvanicus* | *M. montanus* |
| --- | --- | --- | --- | --- | --- | --- | --- | --- |
| Central |  | 0.4% | 0.4% | 0.4% | 0.4% | 0.5% | 0.8% | 0.8% |
| Colorado Plateau | 2.5% |  | 1.3% | 0.3% | 1.0% | 0.5% | 0.8% | 0.8% |
| Island | 2.4% | 0.3% |  | 0.3% | 0.3% | 0.5% | 0.8% | 0.7% |
| Northern | 2.5% | 1.3% | 1.2% |  | 0.9% | 0.5% | 0.8% | 0.7% |
| North Pacific Coast | 2.3% | 0.3% | 1.2% | 0.2% |  | 0.5% | 0.8% | 0.8% |
| Southern | 3.4% | 3.6% | 3.7% | 3.8% | 3.7% |  | 9.9% | 7.8% |
| *M. pennsylvanicus* | 9.9% | 9.3% | 10.2% | 10.1% | 9.9% | 0.8% |  | 0.6% |
| *M. montanus* | 8.5% | 8.3% | 8.6% | 8.9% | 9.0% | 0.7% | 4.7% |  |
|  |  |  |  |  |  |  |  |  |
| *Peromyscus* | *Peromyscus* sp. | *P. keeni* | *P. maniculatus* - East | *P. maniculatus* - West | *P. maniculatus* - Southwest | *P. leucopus* | *P. melanotis* |  |
| *Peromyscus* sp. |  | 0.5% | 0.5% | 0.5% | 0.5% | 0.9% | 1.0% |  |
| *P. keeni* | 3.6% |  | 0.5% | 0.5% | 0.4% | 0.8% | 1.0% |  |
| *P. maniculatus* - East | 4.0% | 3.8% |  | 0.4% | 0.5% | 9.2% | 1.0% |  |
| *P. maniculatus* - West | 3.5% | 3.8% | 3.2% |  | 0.5% | 9.9% | 13.3% |  |
| *P. maniculatus* - Southwest | 3.5% | 2.3% | 3.2% | 3.1% |  | 9.4% | 12.7% |  |
| *P. leucopus* | 10.7% | 10.0% | 0.8% | 0.9% | 0.9% |  | 1.1% |  |
| *P. melanotis* | 13.4% | 13.3% | 12.9% | 1.0% | 1.0% | 14.0% |  |  |
|  |  |  |  |  |  |  |  |  |
| *S. monticola* | Island | Northern | Southern | *S. cinereus* | *S. fumeus* | *S. vagrans* |  |  |
| Island |  | 0.5% | 0.6% | 0.7% | 0.8% | 0.6% |  |  |
| Northern | 4.8% |  | 0.4% | 0.7% | 0.8% | 0.6% |  |  |
| Southern | 5.4% | 1.7% |  | 9.5% | 8.9% | 4.6% |  |  |
| *S. cinereus* | 9.1% | 8.8% | 0.7% |  | 0.7% | 0.7% |  |  |
| *S. fumeus* | 8.4% | 9.0% | 0.8% | 8.4% |  | 0.8% |  |  |
| *S. vagrans* | 5.9% | 5.3% | 0.5% | 8.3% | 8.7% |  |  |  |

**Appendix IX**

Between group net genetic divergences of cyt *b* among refugial and non-refugial Southeast Alaskan populations lineages of *M. longicaudus*, *P. keeni* and *S. monticola*. The number of base differences per site from estimation of net average between groups of sequences is shown. Standard error estimate(s) are shown above the diagonal. All ambiguous positions were removed for each sequence pair. Evolutionary analyses were conducted in mega5.

Data available from the Dryad Digital Repository: https://doi.org/10.5061/dryad.867g4c8

**Appendix X**

Cyt *b* Bayesian skyline plots for hypothesized refugial islands (i) and non-refugial islands (ii; Table 2) for: (a) *M. longicaudus* Island, (b) *P. keeni*, and (c) *S. monticola* Island. The x-axis is from present (left) to past (TMRCA) on the right and is scaled in millions of years, while the y-axis is the log effective population size scaled by generation time. Central line indicates mean change in effective population size through time, with upper and lower lines showing the 95% HDP. Vertical gray bars indicate the LGM (when scale allows) for reference.


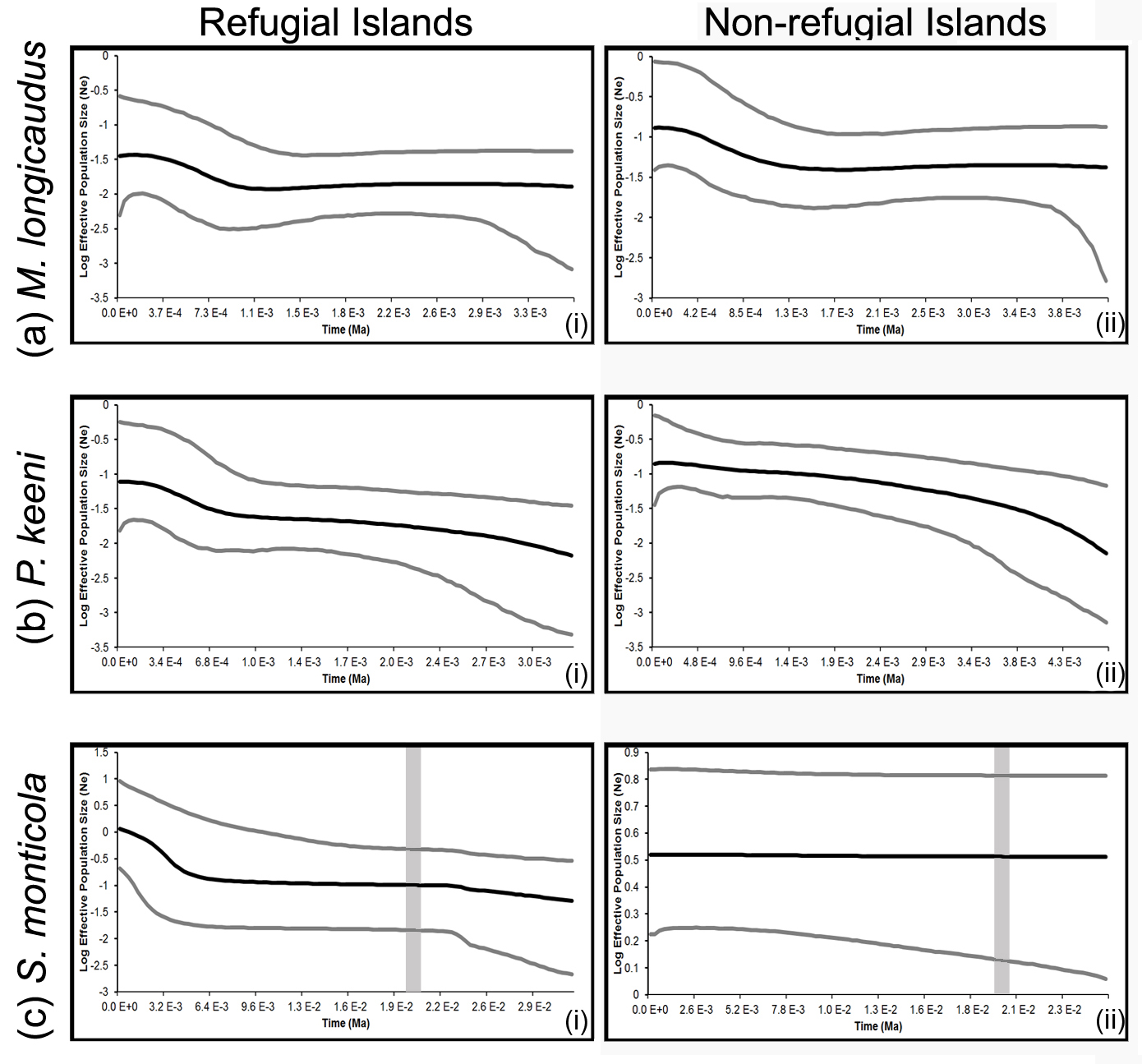

Supplement: Supplementary file 1 [file ECE3-9-1777-s001.docx]
